# Covalent Bonding Between Ir and High-Oxidation State Sb Constrained by Quinoline Scaffolds

**DOI:** 10.1021/acs.inorgchem.5c02934

**Published:** 2025-08-14

**Authors:** Fanji Kong, Christopher K. Webber, Jugal Kumawat, Kevin P. Quirion, Xinrui Ou, Diane A. Dickie, Daniel H. Ess, T. Brent Gunnoe

**Affiliations:** † Department of Chemistry, 2358 University of Virginia, Charlottesville, Virginia 22904, United States; § Department of Chemistry and Biochemistry, 6756 Brigham Young University, Provo, Utah 84604, United States

## Abstract

From the reaction of a high-valent Sb­(V) proligand with a low-valent Ir­(I) precursor in acetonitrile, a bimetallic Sb–Ir complex was isolated in which one of the quinoline groups inverted such that it is *N*-coordinated to Sb and *C*-coordinated to Ir. The new Sb–Ir complex has a unique structure containing the shortest reported Sb–Ir bond (2.51502(18) Å). Our combined experimental and computational studies indicate pronounced covalent character for the Sb–Ir bond. Based on the covalent bonding, the complex more closely resembles Sb­(IV)–Ir­(II) species rather than Sb­(V)–Ir­(I) and thus results in an Ir center with poor π-basicity, particularly toward the position trans to Sb.

## Graphical abstract

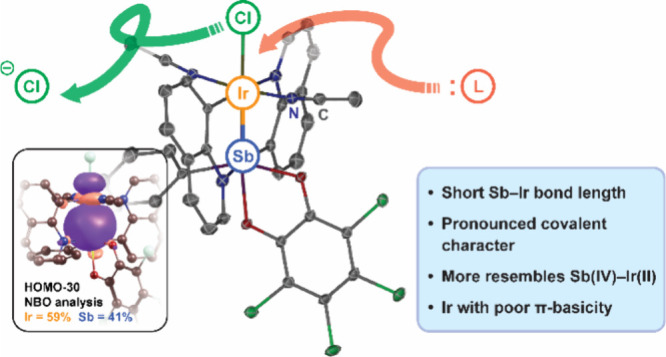

Nitrogen and phosphorus have been widely used in building ligands for transition-metal catalysts due to their well-established steric and electronic properties. In contrast, ligand structures built on heavier pnictogens are less developed and have been primarily confined to trivalent antimony (R_3_Sb) as analogs of monodentate phosphines (R_3_P). There are only 12 published Sb–Ir complexes in the CCDC database,^1–12^ and 9 of them are Ir complexes with a monodentate R_3_Sb ligand.^1–8^ Among the other 3 published structures, one is the coordination of an in-situ-generated [SbF_2_]^+^ cation to an electron-rich bis-Ir­(I) metallomacrocycle^9^ and the other two are based on a bis­(phosphino)­stibine (PSbP) pincer ligand (Scheme 1a).^10,11^ In these studies, a trinuclear cationic Ir–Sb^+^–Ir complex with phosphine arms that involves a three-center four-electron delocalized bond between an Sb cation and two Ir centers was described^10^ and/or a neutral PSbP supported trinuclear bis-Ir­(I) complex that involves a weak interaction between Sb­(III) and one of the Ir­(I) centers.^11^

**Scheme 1:**
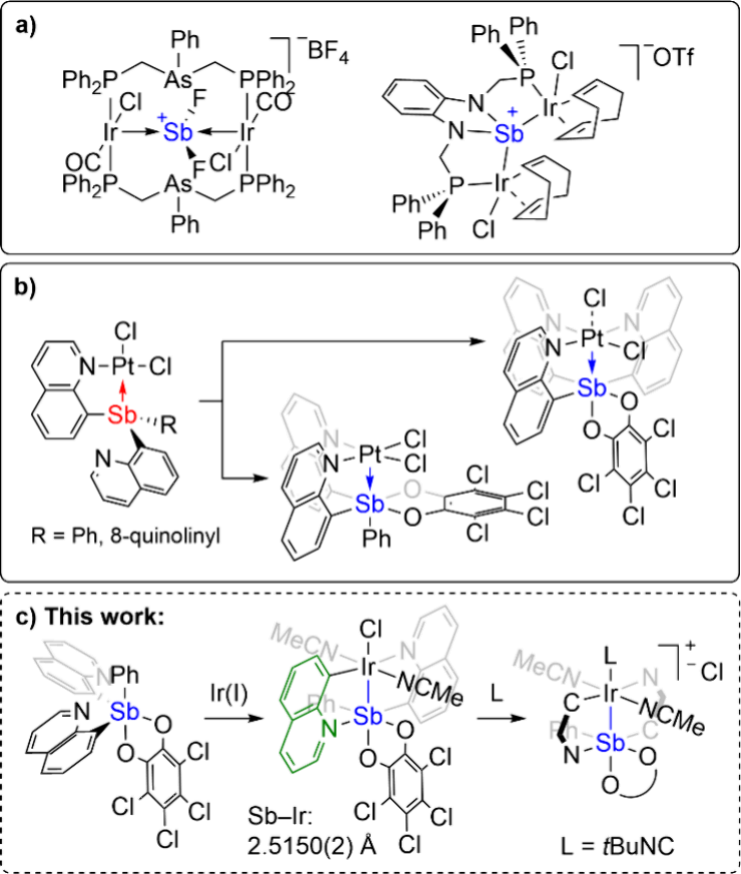
Selected Examples of Heterobimetallic Sb Complexes: (a) Related and Sb–Ir Complexes, (b) Previous study of Sb–Pt Complexes, and (c) Brief Summary of the Current Work

The coordination and redox noninnocent behavior of Sb offers the potential to tune electronic properties of metal centers.^13,14^ The use of L-type donor arms to constrain Lewis acidic coordination sites to a specific location on metals has been demonstrated,^15–21^ and, also, this strategy has been used for incorporating Sb into transition-metal complexes. For example, the Gabbaï group has reported a series of Sb ligands with phosphine arms that offer tunable σ-accepting abilities.^21–35^ More recently, several groups, including ours, have developed Sb ligands that possess chelating nitrogen donors (e.g., quinoline, amine, and imine) that potentially offer alternative coordination environments, compared to antimony–phosphine ligands.^36–42^ In our previous work, we reported the synthesis of Sb–Pt complexes bearing L- or Z-type Sb–Pt interactions by using Sb ligands with quinoline arms (Scheme 1b).^36^ Using different two-electron oxidants as well as a variable number of quinoline groups in the Sb proligand both led to different Sb–Pt bonding modes; chloride transfer between Sb and Pt was observed in the case of using dichloro­(phenyl)-λ^3^-iodane as the oxidant.^36^ More recently, our group has employed a strategy to access an Sb←Rh complex by inhibiting the halide transfer in the reaction of high-valent Sb­(V) proligands with low valent Rh precursors.^43^ Herein, we report the synthesis of a new Sb–Ir complex with covalent bond character/electron pair sharing between Sb and Ir that exhibits a short bond length (Scheme 1c). Natural bond orbitals (NBO) suggest nearly equal contributions from Sb and Ir. The Cl ligand trans to Sb can be readily replaced by isocyanide, while the acetonitrile ligands remain coordinated.

Starting from a previously reported trivalent Q_2_SbPh compound (where Q = 8-quinolinyl),^36^ we isolated an Sb­(V) proligand, Q_2_SbPh­(*o*-chlor) (**1**, *o*-chlor = *o*-chloranil) with 85% yield (Scheme 2). In the crystal structure of **1** (Figure 1), the distances between Sb1 and N1 (2.5621(40) Å) is shorter than the sum of their van der Waals radii (∑*r*_vdW_(Sb,N) = 4.13 Å)^44^ and closer to the sum of covalent radii (∑*r*_c_(Sb,N) = 2.10(5) Å).^45^ This suggests a pnictogen bond between the Sb and the quinoline N1 atom in **1**,^46,47^ which distorts the 5-coordinate Sb toward a 6-coordinate octahedron geometry.

**Figure 1:**
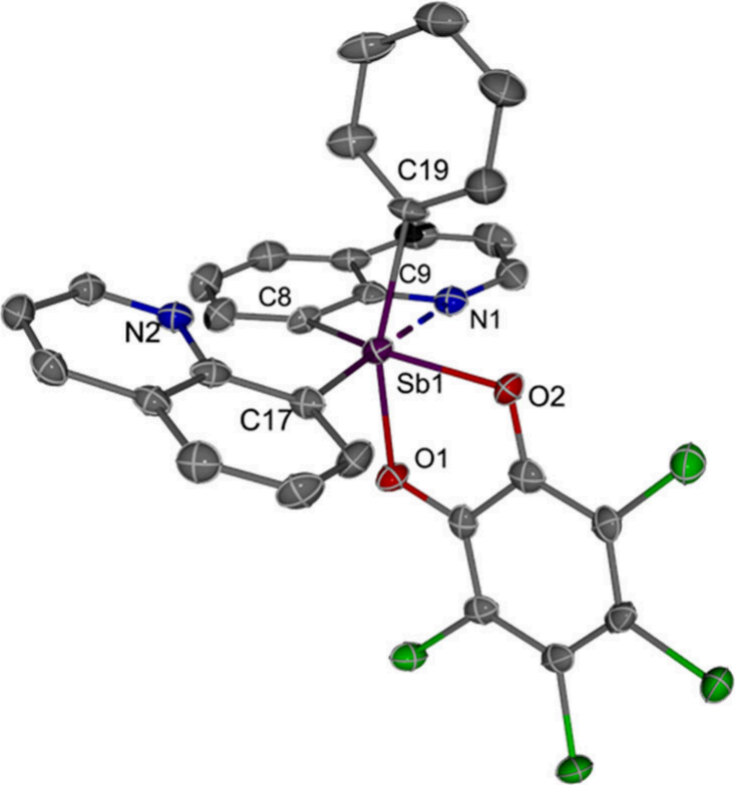
ORTEP of Q_2_SbPh­(*o*-chlor) (**1**). Ellipsoids are drawn at the 50% probability level and hydrogen atoms are omitted for clarity. Selected distances [Å]: Sb1**···**N1, 2.562(4); Sb1–O1, 2.125(3); Sb1–O2, 2.066(3); Sb1–C8, 2.155(4); Sb1–C17, 2.148(4); Sb1–C19, 2.153(5).

**Scheme 2:**
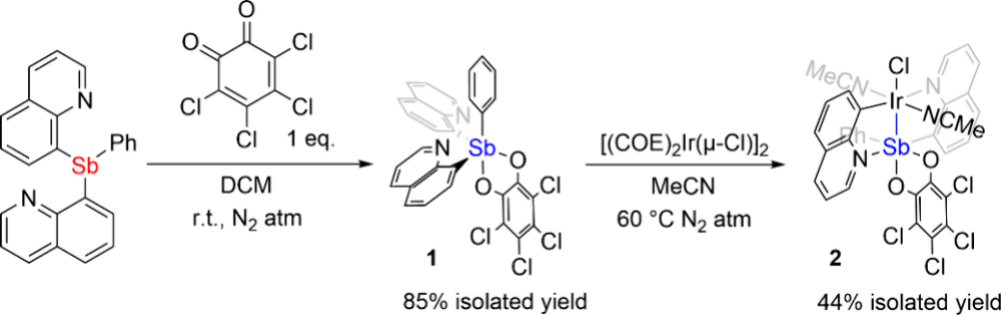
Synthesis of Q_2_SbPh­(*o*-chlor) (**1**) and {Q_2_SbPh­(*o*-chlor)}­Ir­(NCMe)_2_(Cl) (**2**)­^a^

a Legend: *o*-chlor = *o*-chloranil; COE = cyclooctene.

Reacting **1** with 0.5 equiv of [(COE)_2_Ir­(μ-Cl)]_2_ in acetonitrile (MeCN) at 60 °C results in the formation of {Q_2_SbPh­(*o*-chlor)}­Ir­(NCMe)_2_(Cl) (**2**) in 44% isolated yield (Scheme 2). Interestingly, as shown in the solid-state structure (Figure 2), one of the quinoline arms “inverts” for which the C atom bonds to Ir and the N atom bonds to Sb. In addition, the observation of two sets of quinoline proton resonances in the ^1^H NMR spectrum of **2** suggests the quinoline groups are nonsymmetric, which further supports the observed rearrangement of one quinoline arms. We speculate that the initial coordination leads to an intermediate with a weak Sb­(V)←Ir­(I) interaction, followed by insertion of Ir­(I) into the Sb–C bond to form **2**, similar to the reported insertion of Ir or Rh into a B–Ph bond ().^48^ The Sb–Ir bond (2.51502(18) Å) of **2** is shorter than the previously reported shortest Sb–Ir bond (2.545 Å) observed in a 5-coordinate bis­(η^2^-ethylene) Ir­(I) complex carrying two *i*Pr_3_Sb ligands^1^ as well as the other 11 crystal structures in the CCDC database (from 2.558 to 2.997 Å).^2–12^ This short Sb–Ir bond distance in **2** is potentially caused by (a) the covalent bonding interaction between Ir and the high oxidation state Sb (see below) and/or (b) constraint from the rigid quinoline arms.

**Figure 2:**
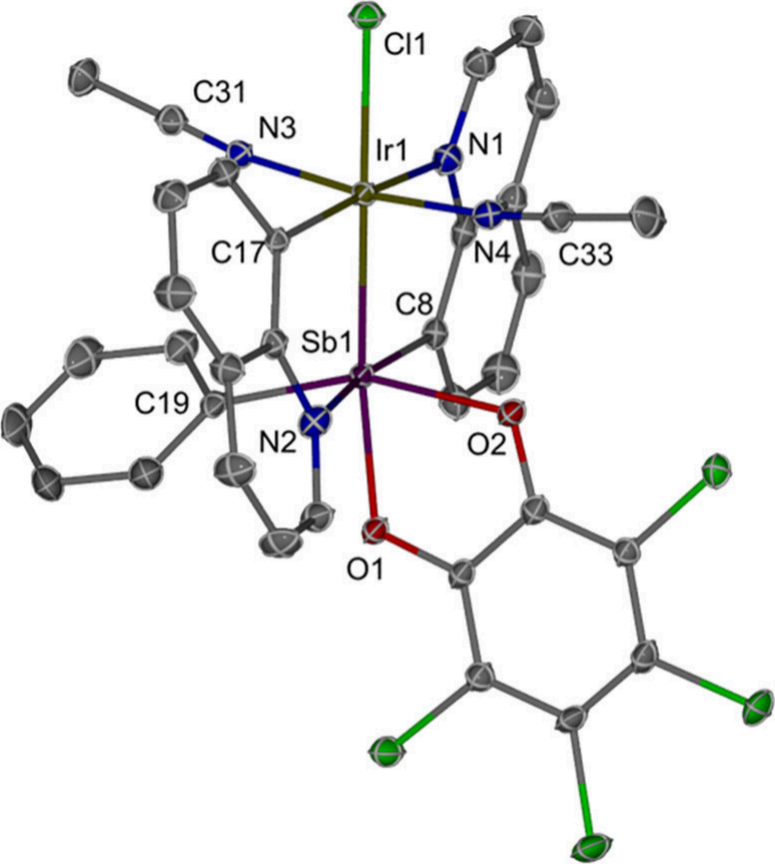
ORTEP of {Q_2_SbPh­(*o*-chlor)}­Ir­(NCMe)_2_(Cl) (**2**). Ellipsoids are drawn at the 50% probability level. Hydrogen atoms and noncoordinating solvents are omitted for clarity. Selected bond lengths [Å]: Sb1–Ir1, 2.51502(18); Ir1–Cl1, 2.4693(6); Ir–N (MeCN–Ir), 1.979(2); and 1.976(2), C≡N (MeCN–Ir) 1.135(3) and 1.140(3).

Based on the 297 crystal structures containing Ir–NCMe fragments,^12^ the Ir–N bond distances (1.979(2) and 1.976(2) Å) for the MeCN ligands in **2** are slightly shorter than average (Ir–N, mean: 2.080 Å), while the C≡N bond lengths (1.135(3) and 1.140(3) Å) are consistent with the reported values (mean: 1.132 Å) and are slightly shorter compared to free acetonitrile (∼1.16 Å). The IR spectrum of **2** shows a weak band at 2302 cm^–1^ for the C≡N stretching, which exhibits a significant blue-shift (49 cm^–1^), compared to free MeCN as a neat liquid (ν_CN_ = 2253 cm^–1^).^49^ Sb–Ir complexes with acetonitrile isotopologues, {Q_2_SbPh­(*o*-chlor)}­Ir­(NCCD_3_)_2_(Cl) (**2-D**) and {Q_2_SbPh­(*o*-chlor)}­Ir­(N^13^CMe)_2_(Cl) (**2-** ^**13**^ **C**), were synthesized to further confirm the identity of the observed weak IR band (). A blue-shift in C≡N stretching frequency is commonly observed when MeCN coordinates in κ^1^-mode to Lewis acids,^50,51^ late transition metals {e.g., Ir­(III)},^52–55^ and metal cations.^56–58^ In contrast, several examples with unchanged or red-shifted ν_CN_ have been observed for more electron-rich low-valent metal centers^59–62^ which is attributed to significant π-backbonding to MeCN.^63^ In complex **2**, the observed 49 cm^–1^ blue-shift in ν_CN_ falls on the lower end of the reported shifts of typical Ir­(III) complexes,^53,54^ but, perhaps importantly, higher than cationic Ir­(I) complexes.^64,65^ M06/def2-SVP density functional theory (DFT) was used to calculate C≡N stretching frequencies in **2**, which are 46 and 30 cm^–1^ blue-shifted compared to the DFT calculated frequency of free MeCN and further supports the experimental measurements and assignments.

As previously reported, Sb offers different coordination modes and bond types to transition-metal centers.^13,25,36,38^ To evaluate possible covalent bond character/electron pair sharing between Ir and Sb, we examined the molecular orbitals, NBOs, and intrinsic bond orbitals (IBO) of **2**. Each of these orbitals can provide a slightly different and nuanced evaluation of possible sharing of an electron pair. Figure 3 shows the DFT-based molecular orbital for the Sb–Ir interaction (HOMO–4), for which visual inspection suggests significant delocalization over both Sb and Ir centers. Consistent with this description, Figure 3 displays the 2D contour map of the Laplacian from quantum theory of atoms in molecules (QTAIM) analysis. This revealed a bonding interaction between Sb and Ir centers with covalent/metallic character at the bond critical point. NBO and IBO analyses show the Ir/Sb bonding contribution as 59%/41% and 62%/36%, respectively. These contribution values, as well as inspection of the molecular orbitals, suggest that the Sb–Ir interaction should be viewed as delocalized with covalent character. This could mean that the Sb is closer to a X-type than a Z-type ligand; however, we did not analyze a hypothetical broken bond to determine if that would generate an electron pair localized on Ir or single electrons on Sb and Ir. Regardless, this covalent bonding is distinct from our previous reported Z-type Sb–Pt complex, in which the Sb–Pt bond contribution was found to be 91% Pt and 5% Sb.^36^ With a relatively covalent bond between the Sb and Ir centers, the structure should be considered to have Sb­(IV)–Ir­(II) oxidation states rather than Sb­(V)–Ir­(I). The proposed Sb­(IV)–Ir­(II) contrasts our previously proposed Sb­(V)–Pt­(II) configuration for a related complex,^36^ but the formally oxidized Ir {i.e., Ir­(II) and not Ir­(I)} and formally reduced Pt {i.e., Pt­(II) and not Pt­(III)} are consistent with periodic trends {e.g., Pt’s Pauling electronegativity (2.28) is slightly greater than that or Ir (2.20)}. The observed C–O and C–C bond lengths and calculated metrical oxidation state of *o*-chloranil in **2** suggest a dianionic form based on the correlation reported by Brown (),^66^ which is also consistent with the proposed Sb­(IV). DFT was used to evaluate the relative energies of the two isomers where the quinoline ligand is inverted (Figure 4), and **2′** was calculated to be 9 kcal·mol^–1^ higher in energy consistent with the experimentally isolated structure.

**Figure 3:**
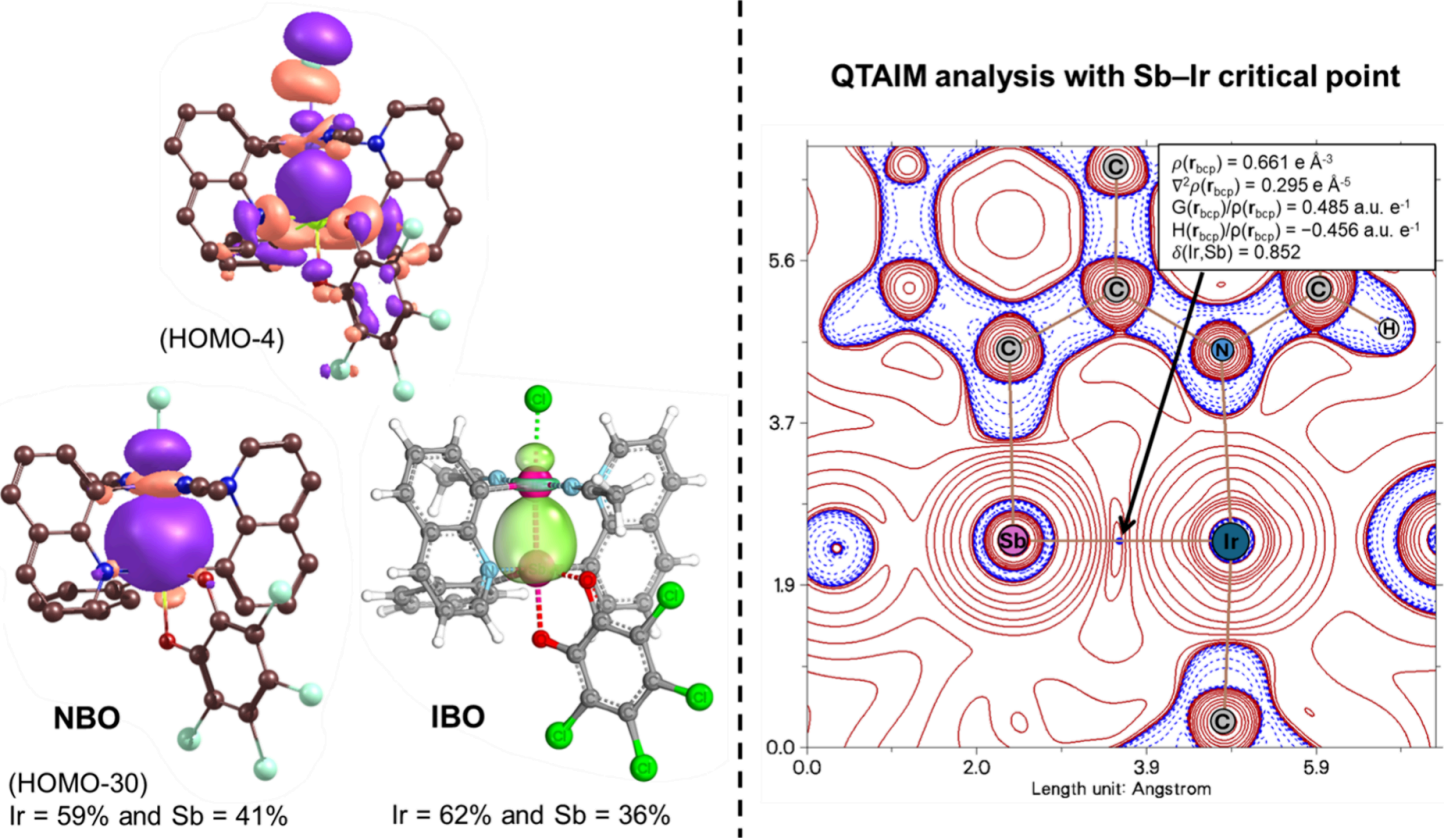
(Left) Molecular orbital, NBO, and IBO the Sb–Ir interaction in **2**. (Right) QTAIM analysis for **2**.

**Figure 4:**
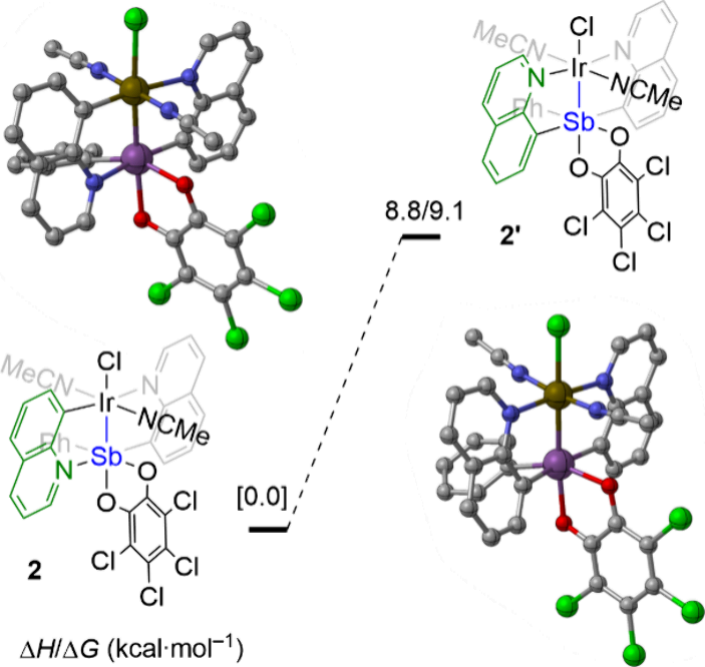
M06/def2-SVP calculated relative enthalpies and Gibbs energies of the isomers for quinoline flip (**2** vs **2′**).

To gain more insight into the electronic properties of the Sb–Ir complex **2**, a strong π-acceptor ligand that is commonly used for IR analysis, CO, was tested for ligand exchange. However, no reaction was found even at elevated temperatures (up to 80 °C). Moving to a stronger σ-donor ligand, *tert*-butyl isocyanide (*t*BuNC), resulted in dissociation of chloride and coordination of *t*BuNC to form ion-pair complex **3** with MeCN ligands remaining coordinated (Scheme 3). In the ^1^H NMR spectrum of **3**, a significant upfield shift (∼1 ppm) of the diagnostic quinoline protons adjacent to the nitrogen suggests the absence of coordinated Cl. Although the exchange reaction cannot reach completion before converting to an unknown complex (), single crystals of **3** were obtained from slow evaporation of the reaction mixture of **2** and **3** (Figure 5). The reaction has been performed in different solvents, and the exchange of *t*BuNC with Cl follows the trend of solvent polarity as expected for which the polar solvents favor the formation of the ion-pair complex **3**.

**Scheme 3:**
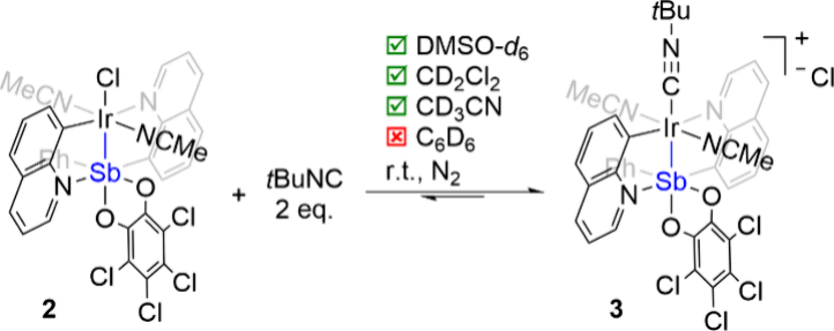
Ligand Exchange Using {Q_2_SbPh­(*o*-chlor)}­Ir­(NCMe)_2_(Cl) (**2**) with *t*BuNC to Form [{Q_2_SbPh­(*o*-chlor)}­Ir­(NCMe)_2_(*t*BuNC)]Cl (**3**)

**Figure 5:**
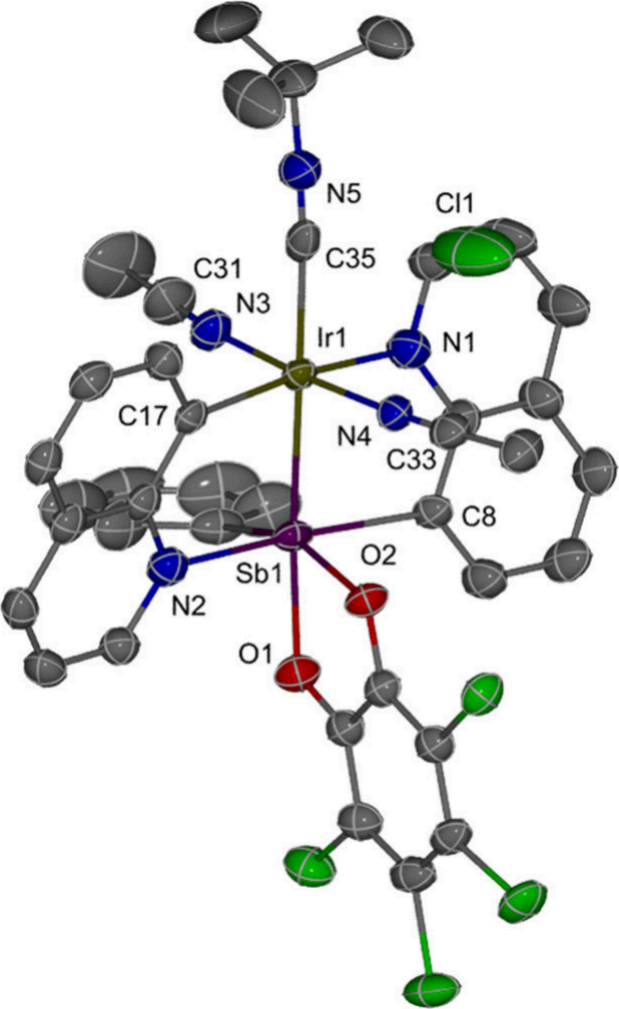
ORTEP of [{Q_2_SbPh­(*o*-chlor)}­Ir­(NCMe)_2_(*t*BuNC)]Cl (**3**). Ellipsoids are drawn at the 50% probability level. Hydrogen atoms and the minor position of the disordered atoms are omitted for clarity. Selected bond lengths [Å]: Sb–Ir, 2.5490(9) and 2.501(10); Ir–C35, 2.085(5) and 2.052(8); C35–N5, 1.104(6); N3–C31, 1.129(6); and N4–C33, 1.124(5). Complex **3** has disordered Sb–Ir atoms, resulting in two separate bond lengths in the structure.

The C≡N stretching frequencies of isocyanides are commonly used to study π-basicity of metal centers, which is a much better probe compared to acetonitrile, due to the stronger π-acidity of isocyanides.^67^ To better understand the ligand exchange of *t*BuNC with chloride and electronic properties of the Ir center, in-situ infrared spectroscopy studies were performed in a solution cell to monitor the reaction of **2** with 2 equiv of *t*BuNC in DMSO-*d*_6_ (Figure 6). Upon the addition of *t*BuNC, a new IR band is observed at 2136 cm^–1^, which is consistent with the reported C≡N stretching frequency of free *t*BuNC (ν_CN_ = 2137 cm^–1^).^68^ As the reaction progressed, a new band at 2197 cm^–1^ increased in intensity, which can be assigned to coordinated *t*BuNC in **3**. For comparison, reported α-dialdimine (*t*BuNC)­Ir­(I) complex exhibit a ν_CN_ at 2124 cm^–1^, while related peroxido (*t*BuNC)­Ir­(III) complex shows a ν_CN_ at 2192 cm^–1^.^69^ Therefore, the observed blue-shifted ν_CN_ in **3** suggests that *t*BuNC bonds through σ-donation, with negligible π-back-donation from the Ir center.

**Figure 6:**
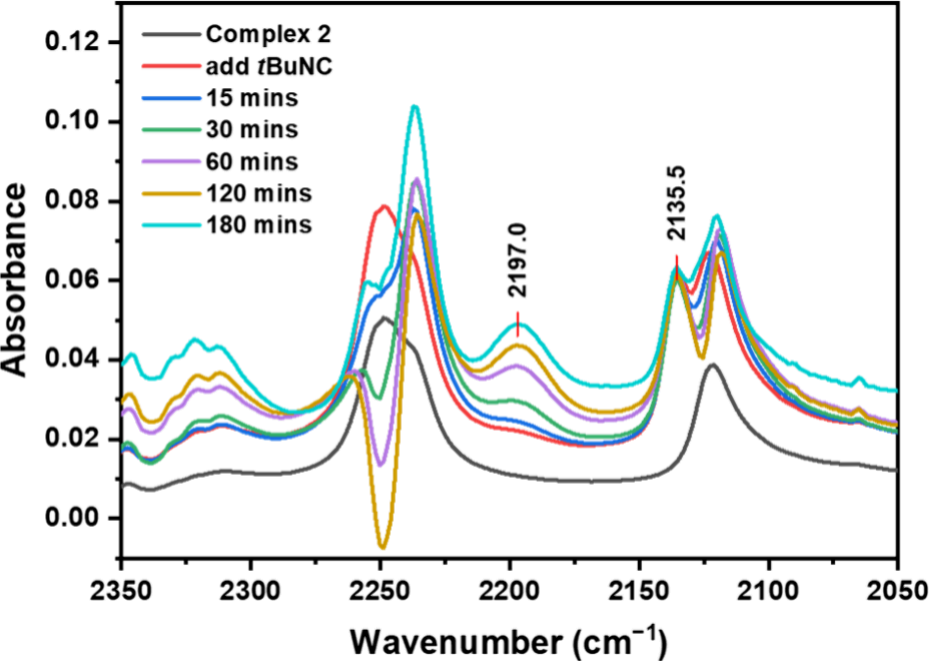
In-situ IR spectra for the reaction of {Q_2_SbPh­(*o*-chlor)}­Ir­(NCMe)_2_(Cl) (**2**) with *t*BuNC in DMSO-*d*_6_.

In summary, moving one group to the left on the periodic table from Pt to Ir results in an increase in Sb–M covalency with almost equal contributions from Sb and Ir (41% and 59%) to the Sb–Ir bond (based on NBO analysis). In contrast, computational analysis of our previously reported Sb–Pt complex reveals an Sb­(V)←Pt­(II) Z-type interaction with 5% Sb and 91% Pt contribution.^36^ This “oxidizing effect” of Sb makes the Ir complexes closer to the Sb­(IV)–Ir­(II) species rather than Sb­(V)–Ir­(I), which is consistent with the observed octahedral geometry and bond lengths. The Sb–Ir interaction seems to suppress Ir π-basicity, especially toward the ligand trans to Sb.

## Supplementary Material





## Notes

Additional experimental procedures and details; NMR and IR spectra; additional single-crystal X-ray structures data; computational details (PDF)
Calculated structure (XYZ)
