# Supplementary material for: Covalent Bonding Between Ir and High-Oxidation State Sb Constrained by Quinoline Scaffolds
Source: Inorg Chem. 2025 Aug 14;64(33):16721–7. doi: 10.1021/acs.inorgchem.5c02934 (PMC12381860; doi:10.1021/acs.inorgchem.5c02934)
Supplement: Supplementary file 1 [file ic5c02934_si_001.pdf]

Supporting Information

Covalent Bonding Between Ir and High-Oxidation State Sb Constrained by Quinoline Scaffolds

Fanji Kong[†,#], Christopher K. Webber[†,#], Jugal Kumawat[§], Kevin P. Quirion[§], Xinrui Ou[†], Diane A. Dickie[†], Daniel H. Ess[§] and T. Brent Gunnoe[†,*]

[†]Department of Chemistry, University of Virginia; Charlottesville, Virginia 22904, United States.

[§]Department of Chemistry and Biochemistry, Brigham Young University; Provo, Utah 84604, United States.

*Corresponding authors. Email: tbg7h@virginia.edu

[#]These authors contributed equally to this work.

Table of Contents

page

1. General Information S3
2. Synthesis and Characterization S4
3. Reaction of Complex **2** with *t*BuNC S9
4. Reaction of Complex **2** with CO S14
5. Metrical Oxidation State of *o*-Chloranil S15
6. Proposed Pathway for Quinoline Inversion S16
7. Attempts for Reacting Sb(III) Proligand to Ir(I) S17
8. NMR Spectra S18
9. IR Spectra S26
10. X-Ray Crystal Structure Data S28
11. Computational Details S30
12. References S32

1. General Information

Di(quinolin-8-yl)-phenyl-λ^3-stibane (**Q_2SbPh**)[1] and $[(COE)_2IrCl]_2$ (COE = cyclooctene)[2] were synthesized based on previous reported procedures. All other chemicals were purchased from commercial sources and used as received. Tetrahydrofuran (THF) and diethyl ether (Et_2O) were dried via a sodium-benzophenone/ketyl still under a dinitrogen atmosphere and stored over activated 4Å molecular sieves inside a glovebox. Pentanes, acetonitrile and methylene chloride were dried using a solvent purification system with activated alumina and stored under activated 4Å or 3Å molecular sieves inside a dinitrogen filled glovebox. Chloroform-*d*, methylene chloride-d_2, dimethyl sulfoxide-d_6, acetonitrile-d_3, and benzene-d_6 were stored over activated 4Å or 3Å molecular sieves inside a glovebox.

All NMR reactions were performed using Wilmad medium wall precision low pressure/vacuum (LPV) NMR tubes. NMR spectra were recorded on a Varian VNMRS 600 MHz, a Bruker Avance III 800 MHz or 600 MHz, or a Bruker Neo Nanobay 400 MHz spectrometer. All reported chemical shifts are referenced to residual ^1H resonances (^1H NMR) or $^{13}C\{^1H\}$ resonances ($^{13}C\{^1H\}$ NMR) of the solvent. ^1H NMR: acetonitrile-d_3 1.94 ppm; chloroform-*d* 7.26 ppm; methylene chloride-d_2 5.32 ppm; dimethyl sulfoxide-d_6 2.50 ppm. $^{13}C\{^1H\}$ NMR: acetonitrile-d_3 1.32 ppm, 118.26 ppm; chloroform-*d* 77.16 ppm; methylene chloride-d_2 53.84 ppm; dimethyl sulfoxide-d_6 39.52 ppm.[3] ^{19}F NMR spectra were referenced to hexafluorobenzene δ −164.9 ppm using an external standard. Infrared spectra were obtained using a Shimadzu IRSpirit-X Compact FT-IR spectrophotometer. Elemental analyses were performed by the University of Virginia Chemistry Department Elemental Analysis Facility using a Perkin-Elmer CHNS-O series II analyzer.

2. Synthesis and Characterization

***Note*: During all synthesis, no uncommon hazards are noted.**

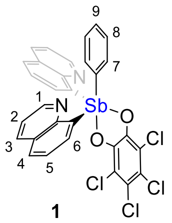

1

Q_2SbPh(*o*-chlor)

Q_2SbPh(*o*-chlor) (1). To a solution of Q_2SbPh (175 mg, 0.38 mmol) in 15 mL of dried DCM, *o*-chloranil (97 mg, 0.40 mmol) was added at room temperature under dinitrogen atmosphere. The color of the solution was changed from red orange to light yellow. After stirring overnight, the mixture was vacuumed to dryness, and ~2 mL of THF was added to dissolve the resulting solid crude product. Then, pentanes were slowly added to precipitate a pale-yellow solid. The solid was collected by vacuum filtration and washed with pentanes to yield a pure product (230 mg, 85% isolated yield). Single crystals for X-ray diffraction can be obtained via either slow evaporation of a DCM solution of **1**, or vapor diffusion of pentanes or diethyl ether into a chloroform or 1,2-dichloroethane solution of **1**. ^{1}H NMR (400 MHz, $CDCl_3$, ppm) δ=8.62 (dd, $^3J_{H,H}$ = 4 Hz, $^4J_{H,H}$ = 2 Hz, 2H; *H1*), 8.45 (dd, $^3J_{H,H}$ = 7 Hz, $^4J_{H,H}$ = 1 Hz, 2H; *H6*), 8.19 (dd, $^3J_{H,H}$ = 8 Hz, $^4J_{H,H}$ = 2 Hz, 2H; *H4*), 7.96 – 7.88 (m, 4H; H_3 and phenyl-*H7*), 7.68 (dd, $^3J_{H,H}$ = 8, 7 Hz, 2H; *H2*), 7.36 (dd, $^3J_{H,H}$ = 8, 4.4 Hz, 2H; *H5*), 7.27 (t, 3H; phenyl-*H8* and *H9*, overlapped with residual chloroform peak.); ^{13}C{^{1}H} NMR (201 MHz, $CDCl_3$, ppm) δ=149.0, 148.6, 148.4, 146.8, 144.1, 136.7, 135.7, 133.9, 130.3, 129.4, 128.7, 128.6, 127.9, 122.0, 119.3, 115.9; IR(KBr, cm^{-1}): $\tilde{v}$=1248 (s), 978 (s)(C–O); elemental analysis calcd (%) for $C_{30}H_{17}Cl_4N_2O_2Sb{\cdot}0.18CH_2Cl_2$: C 50.60, H 2.44, N 3.91; found: C 50.59(17), H 2.31(2), N 3.84(2) Standard deviations were calculated from three independent runs.

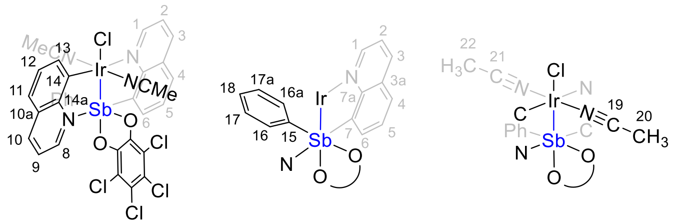

{Q_2SbPh(*o*-chlor)}Ir(NCMe)$_2$(Cl) (2). To a pressure tube with proligand **1** (100 mg, 0.14 mmol) and [(COE)$_2$IrCl]$_2$ (64 mg, 0.07 mmol), 10 mL of dried MeCN was added inside a dinitrogen filled Glovebox. The pressure tube was then sealed and heated in a 60 °C oil bath. All the solids dissolved within 30 mins at 60 °C, and then yellow precipitates formed. After 1 hour, the pressure tube was sent back into the glovebox and the suspension was concentrated to about 2–3 mL via vacuum. The solid was collected by vacuum filtration and washed with minimum amount of THF (~1 mL) to yield the pure product as a bright yellow solid (63 mg, 44% isolated yield). The product can be further purified via reprecipitation or bulk recrystallization using MeCN and Et_2O if needed. The product is relatively air stable. ^{1}H NMR (600 MHz, DMSO-d_6, ppm) δ=11.06 (d, $^3J_{H,H}$ = 5 Hz, 1H; *H1*), 9.58 (d, $^3J_{H,H}$ = 7 Hz, 1H; *H13*), 8.78 (d, $^3J_{H,H}$ = 8 Hz, 1H; *H3*), 8.72 (d, $^3J_{H,H}$ = 5 Hz, 1H; *H8*), 8.67 (d, $^3J_{H,H}$ = 8 Hz, 1H; *H10*), 8.18 (d, $^3J_{H,H}$ = 8 Hz, 1H; *H6*), 8.10 (d, $^3J_{H,H}$ = 7 Hz, 1H; *H4*), 7.86 (dd, $^3J_{H,H}$ = 8, 5 Hz, 1H; *H2*), 7.81 (q, $^3J_{H,H}$ = 8 Hz, 2H; *H11 and H5*), 7.70 (dd, $^3J_{H,H}$ = 8, 5 Hz, 1H; *H9*), 7.55 (t, $^3J_{H,H}$ = 8 Hz, 1H; *H12*), 7.07 (t, $^3J_{H,H}$ = 7 Hz, 1H; phenyl-*H18*), 7.02 (t, $^3J_{H,H}$ = 8 Hz, 2H; phenyl-*H17* and *H17a*), 6.66 (d, $^3J_{H,H}$ = 8 Hz, 2H; phenyl-*H16* and *H16a*), 2.33 (s, 3H; C(*H22*)$_3$CN), 1.63 (s, 3H; C(*H20*)$_3$CN). ^{13}C{^{1}H} NMR (201 MHz, DMSO-d_6, ppm) δ=157.3 (*C1*), 148.8 (*C7a*), 146.8 (*C8*), 146.43 (*C15*), 146.41 (*C14a*), 146.2 (*C7*), 145.6 (*C13*), 142.8 (*C10*), 140.8 (*C3*), 136.2 (*C4*), 131.6 (phenyl-*C16 and C16a*), 130.6 (*C6*), 130.2 (*C3a*), 130.1 (*C14*), 129.0 (*C10a*), 127.9 (phenyl-*C18*), 127.8 (phenyl-*C17 and C17a*), 127.4 (*C5*), 127.1 (*C12*), 124.2 (*C11*), 122.6

(*C2*), 120.6 (*C9*), 117.3 (br s, *C*Cl on *o*-chlor), 116.7 (CH_3(*C21*)N), 116.4 (CH_3(*C19*)N), 115.1 (br s, *C*Cl on *o*-chlor), 114.6 (br s, *C*Cl on *o*-chlor), 112.7 (br s, *C*Cl on *o*-chlor), 3.1 ((*C20*)H_3*C*N), 1.9 ((*C22*)H_3*C*N). *Note:* two peaks buried around 148.5 ppm likely belongs to the o-chloranil *C*–O (see **Figure S12**). The *o*-chloranil carbons were broadened and not clearly observed likely due to the short free induction decay caused by the quadrupole atoms. IR(KBr, cm^{-1}): $\tilde{v}$=2302 (w)(C≡N), 1249 (s), 975 (s)(C–O); elemental analysis calcd (%) for $C_{34}H_{23}Cl_5N_4O_2SbIr$: C 40.40, H 2.29, N 5.54; found: C 40.01, H 2.11, N 5.48.

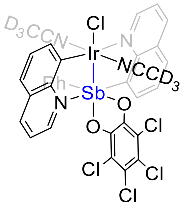

{Q_2SbPh(*o*-chlor)}Ir($NCCD_3$)$_2$(Cl) (2-D). The complex can be made using the same procedure as complex **2** using CD_3CN with 34% isolated yield (25 mg) from a 50 mg scale reaction in 5 mL CD_3CN. IR(KBr, cm^{-1}): $\tilde{v}$=2312 (w)(C≡N). In the ^{1}H NMR spectrum, no proton resonances were observed at 2.33 and 1.63 ppm.

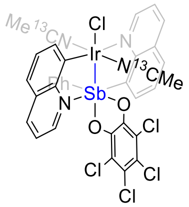

{Q_2SbPh(*o*-chlor)}Ir(N^{13}CMe)$_2$(Cl) (2-^{13}C). Due to the cost of Me^{13}CN, the synthesis was performed using a small amount of solvent (1 mL), and the reaction solution was reused to isolate multiple batches. The commercially purchased Me^{13}CN was passed through pre-dried neutral alumina and dried over 4Å molecular sieves before use. The first batch was done on a 30 mg scale. After heating at 60 °C for 1 hour, the solid was collected via filtration. The filtrate was collected and reused for the next batch (20 mg scale). The insoluble solids were combined from several batches, then washed with THF (1 mL three times) to remove the unreacted free ligand and side-products. The remaining solid was collected as the pure product (~5 mg). *Note:* Heating with undried solvent during the synthesis leads to the decomposition of product. THF also dissolved some of the products during the washing process. Selected ^{1}H NMR (600 MHz, DMSO-d_6, ppm) δ=2.33 (d, $^2J_{C,H}$ = 10 Hz, 3H; CH_3^{13}CN–Ir), 1.63 (d, $^2J_{C,H}$ = 10 Hz, 3H; CH_3^{13}CN–Ir). ^{13}C{^{1}H} NMR (201 MHz, DMSO-d_6, ppm) δ 116.7 (CH$_3$^{13}*C*N–Ir), 116.4 (CH$_3$^{13}*C*N–Ir). IR(KBr, cm^{-1}): ṽ=2255 (w)(C≡N).

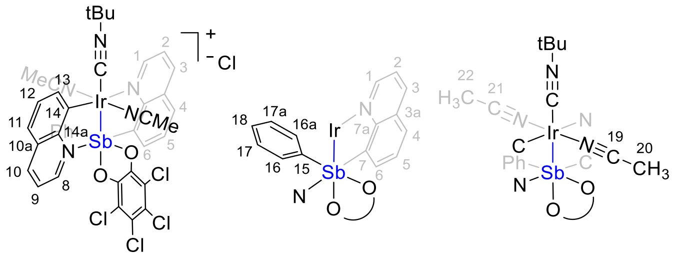

[{Q_2SbPh(*o*-chlor)}Ir(NCMe)$_2$(*t*BuNC)]Cl (3). As described in the main paper, complex **3** was made *in situ* under dinitrogen atmosphere by adding 2.0 eq. of tBuNC (2.2 µL, 0.020 mmol) to a solution of complex **2** (10 mg, 0.010 mmol) in 0.5 mL of DMSO-d_6, CD_3CN, or CD_2Cl_2 at room temperature or 40 °C. Single crystals for X-ray diffraction can be obtained via slow evaporation. ^{1}H NMR (600 MHz, DMSO-d_6, ppm) δ=10.03 (dd, $^3J_{H,H}$ = 5 Hz, $^4J_{H,H}$ = 2 Hz, 1H; *H1*), 8.96 (dd, $^3J_{H,H}$ = 8 Hz, $^4J_{H,H}$ = 2 Hz, 1H; *H13*), 8.77 (dd, $^3J_{H,H}$ = 5 Hz, $^4J_{H,H}$ = 2 Hz, 1H; *H8*), 8.75 (dd, $^3J_{H,H}$ = 8 Hz, $^4J_{H,H}$ = 2 Hz, 1H; *H3*), 8.59 (dd, $^3J_{H,H}$ = 7 Hz, $^4J_{H,H}$ = 1 Hz, 1H; *H10*), 8.22 (dd, $^3J_{H,H}$ = 8 Hz, $^4J_{H,H}$ = 2 Hz, 1H; *H6*), 8.13 (dd, $^3J_{H,H}$ = 7 Hz, $^4J_{H,H}$ = 2 Hz, 1H; *H4*), 7.99 (dd, $^3J_{H,H}$ = 8 Hz, $^4J_{H,H}$ = 1 Hz, 1H; *H11*), 7.94 (dd, $^3J_{H,H}$ = 8 Hz, $^4J_{H,H}$ = 5 Hz, 1H; *H2*), 7.87 (t, $^3J_{H,H}$ = 7 Hz, 1H; *H5*), 7.80 (dd, $^3J_{H,H}$ = 8 and 5 Hz, 1H; *H9*), 7.63 (t, $^3J_{H,H}$ = 8 Hz, 1H; *H12*), 7.12 (t, $^3J_{H,H}$ = 7 Hz, 1H; phenyl-*H18*), 7.07 (t, $^3J_{H,H}$ = 7 Hz, 2H; phenyl-*H17* and *H17a*), 6.71 (d, $^3J_{H,H}$ = 7 Hz ,2H; phenyl-*H16* and *H16a*), 2.43 (s, 3H; C(*H22*)$_3$CN), 1.87 (s, 9H, *t*Bu), 1.69 (s, 3H; C(*H20*)$_3$CN).

3. Reaction of Complex 2 with *t*BuNC

Adding two equivalents of *tert*-butyl isocyanide (*t*BuNC) into a DMSO-d_6 solution of complex **2**, resulted in dissociation of chloride and coordination of *t*BuNC to form the ion-pair complex **3**. The reaction of **2** with *t*BuNC in DMSO-d_6 reveals an immediate reaction at room temperature, the **2**:**3** ratio reaches 1:3 after 4 hours. Performing the reaction at 40 °C for 1 hour resulted in a ~1:9 ratio of **2**:**3**. The reaction of **2** with *t*BuNC has been performed at variant temperatures in different solvents including DMSO-d_6, C_6D_6, CD_2Cl_2, and CD_3CN (**Table S1**, **Figure S1**). No reaction was observed when using C_6D_6 as the solvent even at high temperature (80 °C). The non-polar nature of C_6D_6 potentially inhibits the formation of a cationic Ir complex. Upon heating in CD_2Cl_2 at 40 °C for 1 day, the **2**:**3** ratio only reaches a ratio of ~1:0.7. Despite the poor solubility of complex **2** in MeCN, **3** is formed by adding *t*BuNC into a suspension of **2** in CD_3CN with a **2**:**3** ratio of ~1:8 in solution after 4 hours at room temperature. However, > 70% of the remaining solid in the NMR tube was found to be complex **2** (measured in CD_2Cl_2). As shown in **Figure S1**, the integration of the coordinated MeCN ligands remains unchanged as 3-to-1 versus the quinoline protons in both **2** and **3**, which suggests no dissociation of the MeCN ligands.

Table S1. Summary of **3**-to-**2** ratio in the reaction of **2** with *t*BuNC.[a]

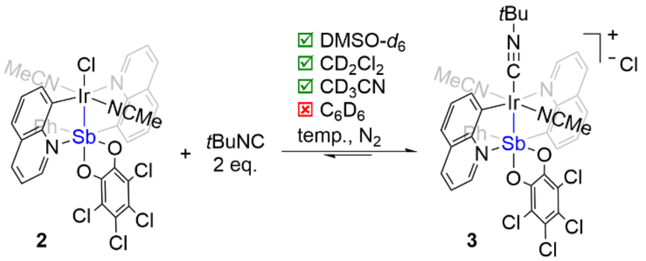

Entry	Solvent	Temperature	Time	**3**-to-**2** Ratio
1	DMSO-d_6	r.t.	<5 min[b]	0.3

2	DMSO-d_6	r.t.	1 h	0.9
3	DMSO-d_6	r.t.	4 h	3.0
4	DMSO-d_6	40 °C	1 h	9.3
5	DMSO-d_6	40 °C	overnight	15.3[c]
6	DMSO-d_6	70 °C	2 h	n/a[c]
7	CD_3CN[d]	r.t.	4 h	7.6[e]
8	CD_2Cl_2	40 °C	1 day	0.7
9	C_6D_6	r.t. to 80 °C	overnight	N.R.

[a] Condition: complex **2** (10 mg, 0.0099 mmol); *t*BuNC (2.2 μL, 0.0198 mmol); solvent (0.5 mL). [b] Taking ^{1}H NMR right after addition of *t*BuNC. [c] Unknown new species formed. [d] Complex **3** did not completely dissolve. [e] Ratio in solution phase.

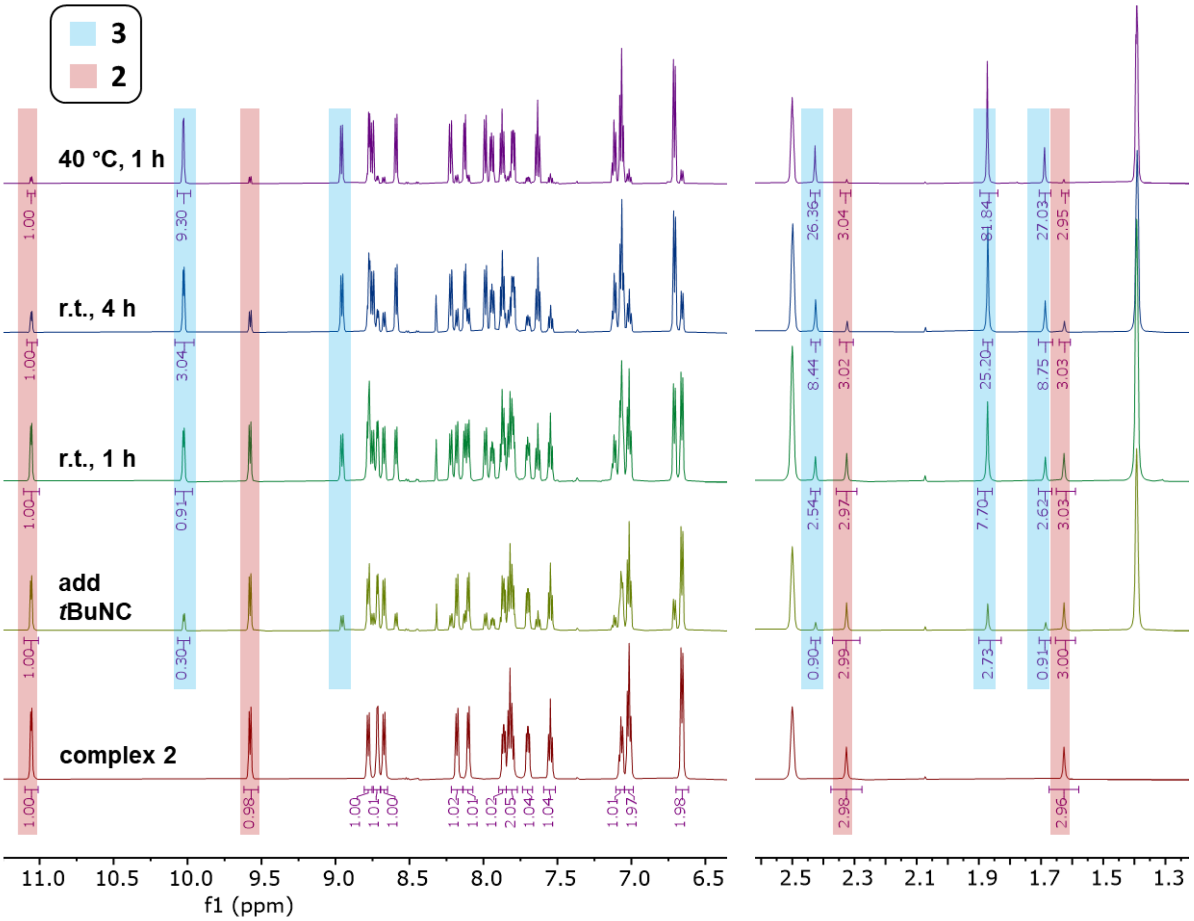

Figure S1. *In situ* ^{1}H NMR spectra of reacting {Q_2SbPh(*o*-chlor)}Ir(NCMe)$_2$(Cl) (**2**) with 2.0 eq. tBuNC to form [{Q_2SbPh(*o*-chlor)}Ir(NCMe)$_2$(*t*BuNC)]Cl (**3**) (600 MHz, DMSO-d_6).

Using ^{1}H NMR spectroscopy to monitor the reaction of **2** with *t*BuNC in DMSO-d_6 reveals an immediate reaction at room temperature to from complex **3**. Heating the reaction mixture at 40 °C for 1 h lead to over 90% conversion of **2** to **3**. However, prolonged heating at 40 °C overnight or at 70 °C for 2 hours led to the conversion of complex **3** to unknown species (**Figure S2**). During which, the residual complex **2** in the mixture remained observable in the *in situ* ^{1}H NMR spectra, indicating that the conversion of **3** to unknown species starts before the full conversion of **2** to **3**. Further study into this reactivity is in progress.

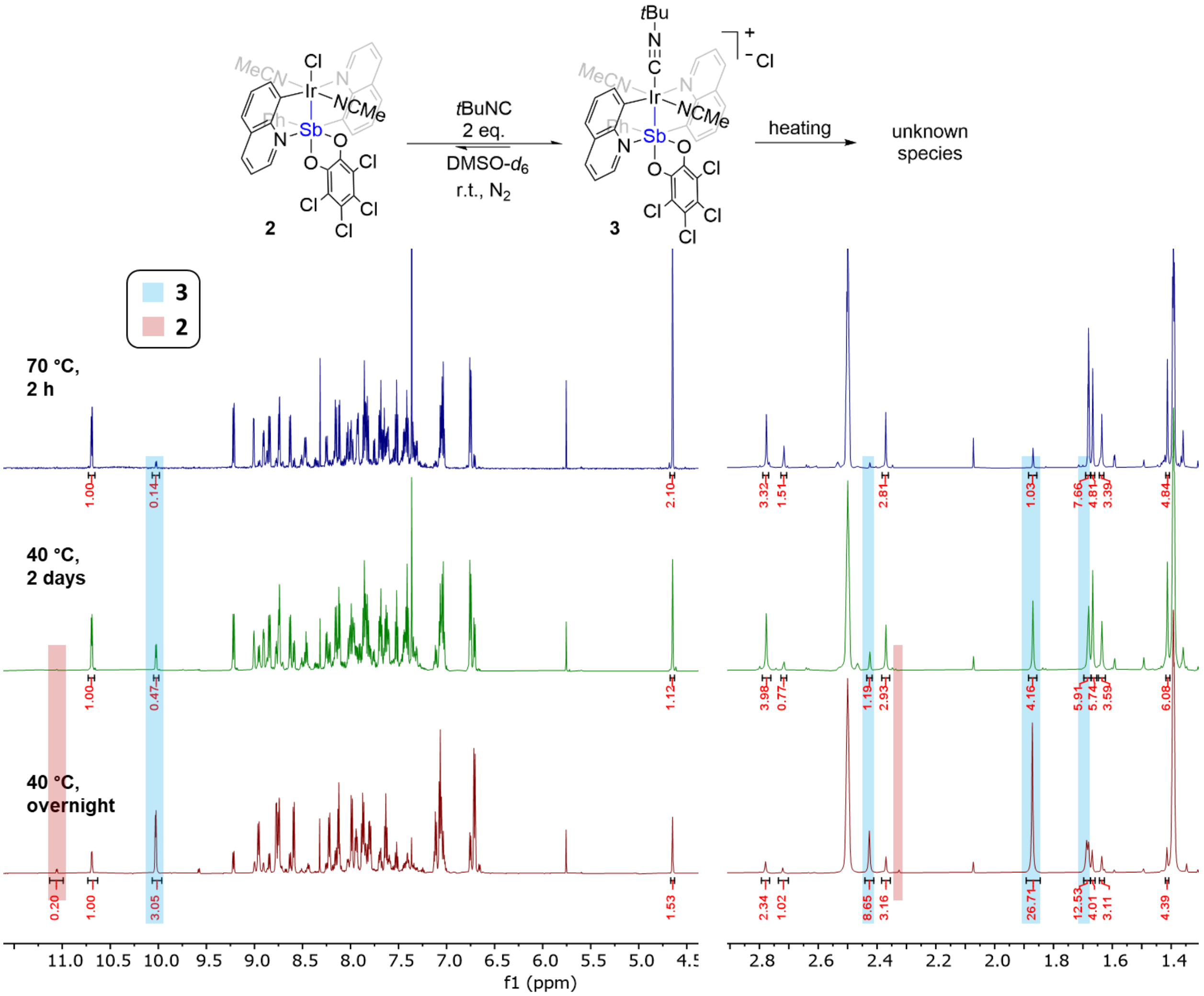

Figure S2. *In situ* ^{1}H NMR spectra of the conversion of [{Q_2SbPh(*o*-chlor)}Ir(NCMe)$_2$(*t*BuNC)]Cl (**3**) to unknown species (600 MHz, DMSO-d_6).

Sodium tetrakis[3,5-bis(trifluoromethyl)phenyl]borate (NaBArF) was added after *t*BuNC to drive the reaction forward via halide abstraction. However, the ^{1}H NMR spectra show almost no difference between the reaction with and without the NaBArF (**Figure S3**). Therefore, it is unclear whether chloride has been successfully abstracted.

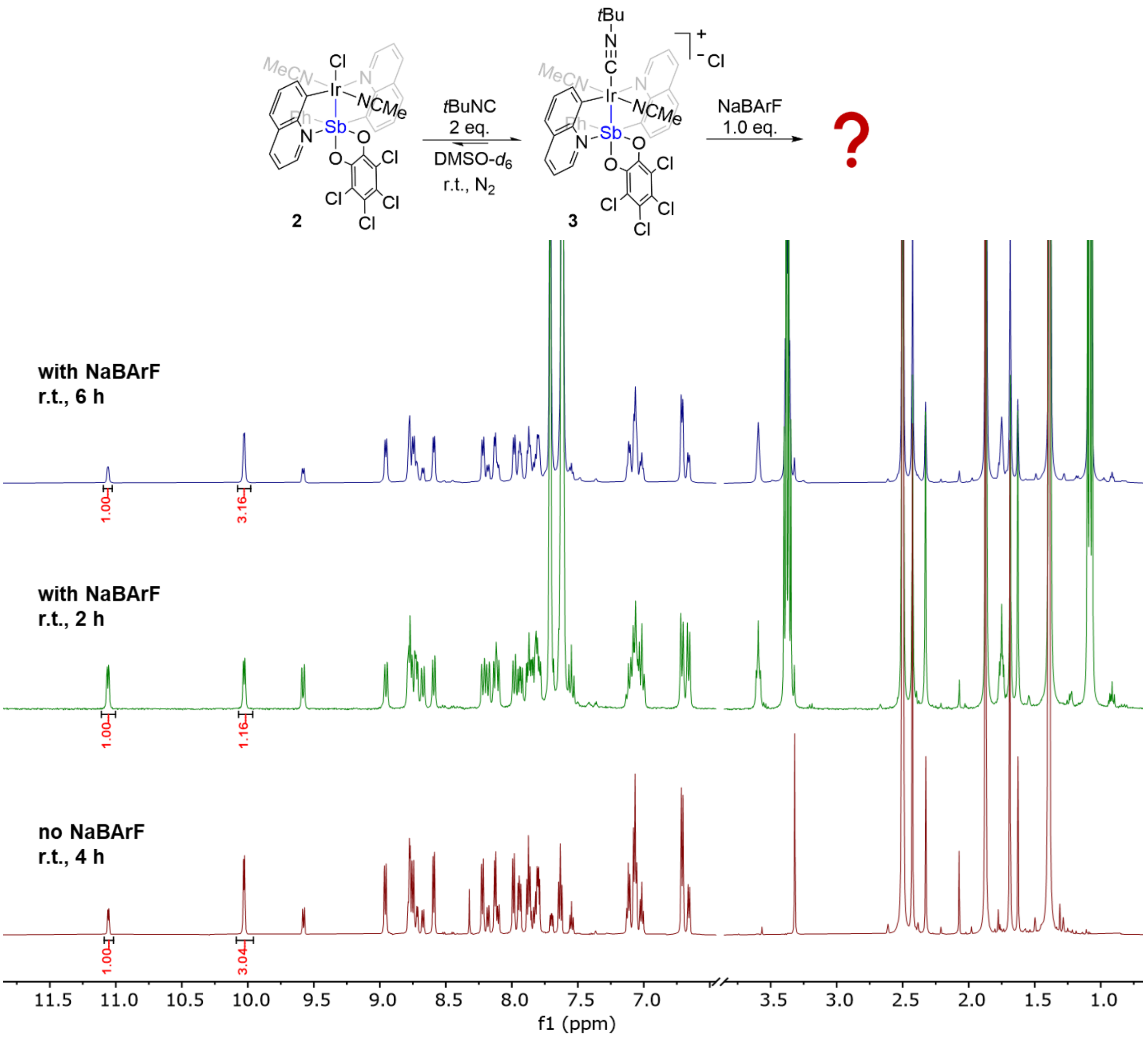

Figure S3. ^{1}H NMR spectra of reacting complex **2** with 2.0 eq. *t*BuNC to form complex **3** with and without 1.0 eq. of NaBArF (400 or 600 MHz, DMSO-d_6).

We have also tested halide abstraction using NaBArF in non-coordinating solvent (C_6D_6), however, the reaction resulted in conversion to multi-products immediately (**Figure S4**). Based on the chemical shift of the most downfield peaks (below 9.5 ppm), there is either no ligand trans to Sb, or the quinoline arms are the Ir center.

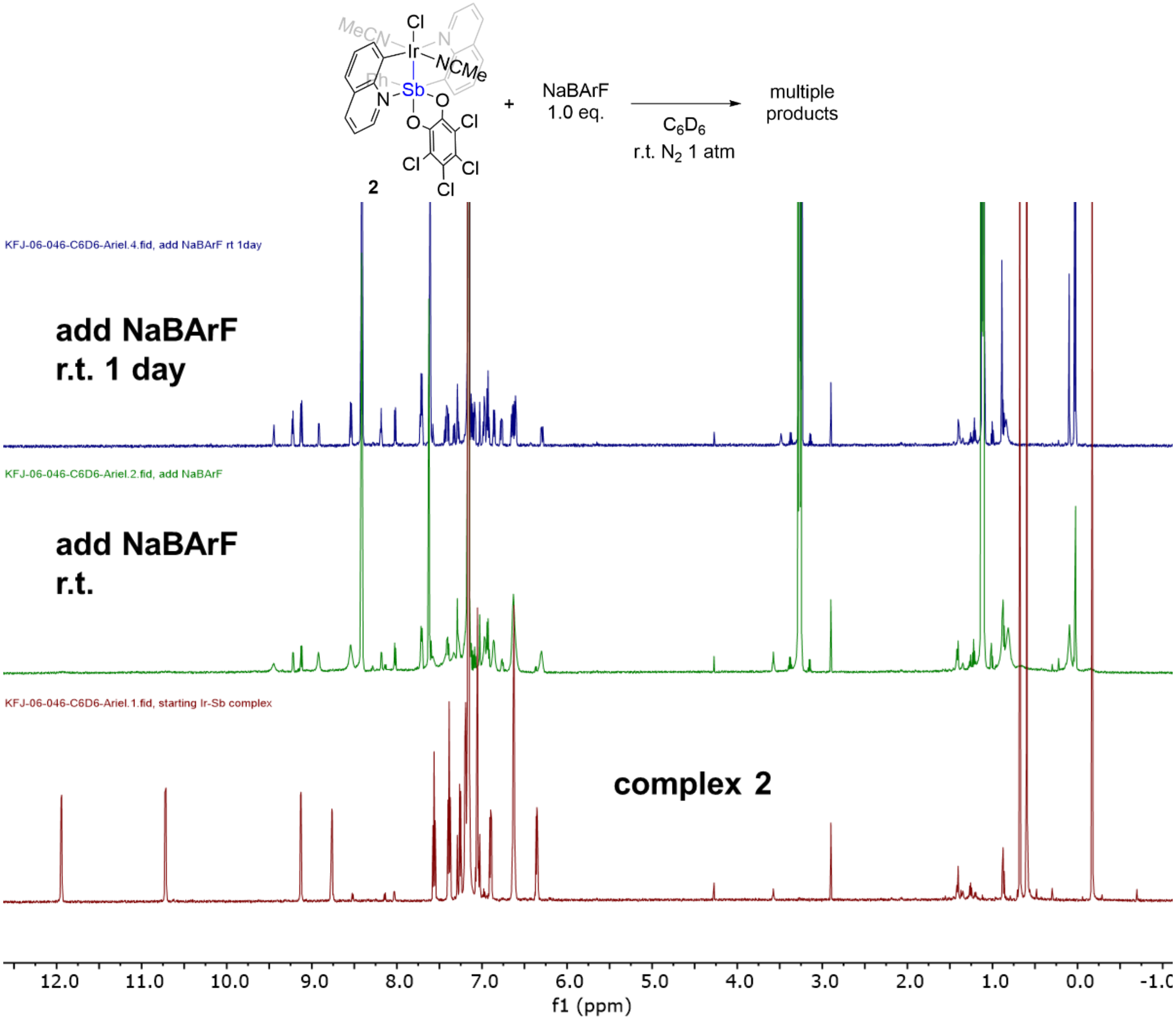

Figure S4. *In situ* ^{1}H NMR spectra of reacting complex **2** with 1.0 eq. of NaBArF (600 MHz, C_6D_6).

4. Reaction of Complex 2 with CO

To a solution of complex **2** in DMSO-d_6 in a J-Young tube, 20 psig of CO was charged and kept at r.t. or heated at 60 or 80 °C. As a result, no reaction has been observed for exchange of CO to the Cl ligand on Ir (**Figure S5**).

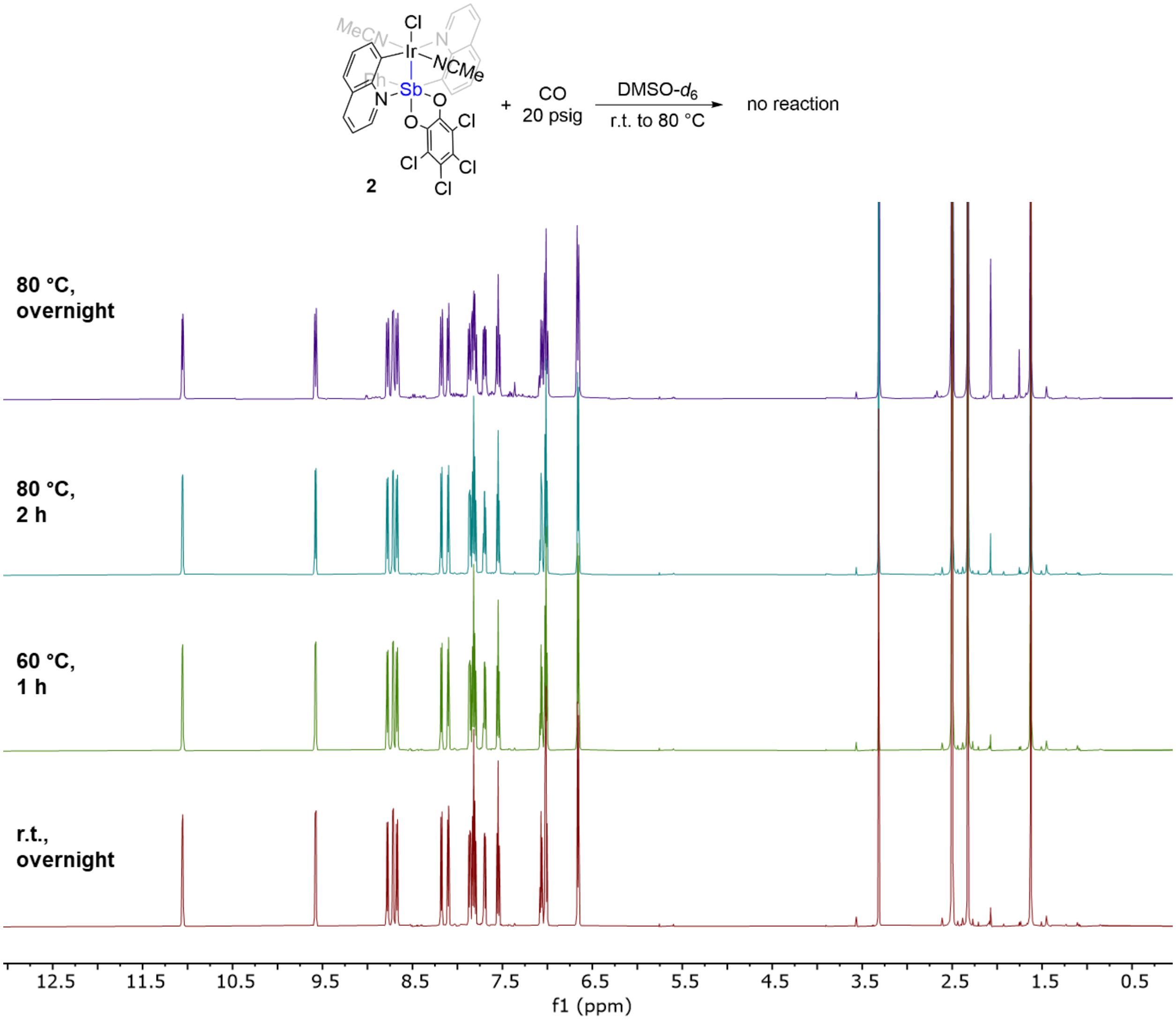

Figure S5. ^{1}H NMR spectra of reacting {Q_2SbPh(*o*-chlor)}Ir(NCMe)$_2$(Cl) (**2**) with 20 psig CO (400 or 600 MHz, DMSO-d_6).

5. Metrical Oxidation State of *o*-Chloranil

Catecholate can potentially act as a redox non-innocent ligand, forming metal complexes in the monoanionic semiquinone or neutral quinone forms instead of the dianionic form. A study by Brown has demonstrated a correlation between bond lengths and ligand oxidation state, and proposed a systematic "metrical oxidation state" (MOS) for better represent the apparent oxidation state of the ligand.[4] **Table S2** listed the calculated MOS values, along with the C–O and C–C bond lengths of the *o*-chloranil moiety in complexes **1**, **2** and **3** used for the calculations. For both Sb–Ir complexes **2** and **3**, the calculated MOS values are nearly equal to −2, suggesting that the *o*-chloranil moiety is better classified as the typical dianionic form, which is consistent with our proposed Sb(IV) formal oxidation state. Proligand **1** has a MOS value of −1.64(16), which slightly differs from the typical dianionic form, though still distinct from the semiquinone form.

Table S2. Summary of bond lengths of the *o*-chloranil moiety and the calculated metrical oxidation state (MOS).[a]

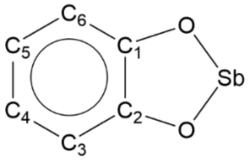

Bond length (Å)	Complex **1**	Complex **2**	Complex **3**
Avg. of C–O	1.3115	1.3255	1.318
C1–C2	1.427	1.415	1.408
Avg. of C2–C3 & C1–C6	1.3955	1.3855	1.393
Avg. of C3–C4 & C5–C6	1.4005	1.4015	1.3905
C4–C5	1.381	1.387	1.371
Calc. MOS	−1.64(16)	−1.79(12)	−1.79(16)

[a] The MOS were calculated using the MOS calculator Excel spreadsheet in the published by Seth N. Brown in *Inorg. Chem.* **2012**, *51*, 1251−1260.

6. Proposed Pathway for Quinoline Inversion

The insertion of transition metals into Sb–Cl bonds have been reported; however, the insertion of transition metals into Sb–C bond is uncommon. A related process is Ir(I) or Rh(I) insertion into a B–Ph bond via oxidative addition reported by the Ozerov group (**Scheme S1a**).[5] One difference between B and Sb is how to consider the nature of Ir–BR_2 vs Ir–SbR_4 bond, which will likely affect the charge on Ir and the insertion mechanism (*e.g.*, potential radical vs anion transfer). We speculated that the inversion might be initiated by formation of an intermediate with a weak Sb(V)←Ir(I) interaction, similar to the reported case of Rh insertion into B–Ph bond. Subsequently, insertion of Ir(I) into the Sb–C bond likely occurs, leading to the formation of **2** with an inverted quinoline arm (**Scheme S1b**). This is also consistent with the speculated pathway of Ni insertion into Sb–C bond reported by Gabbaï group.[6] Further studies to better understand this insertion process are currently in progress.

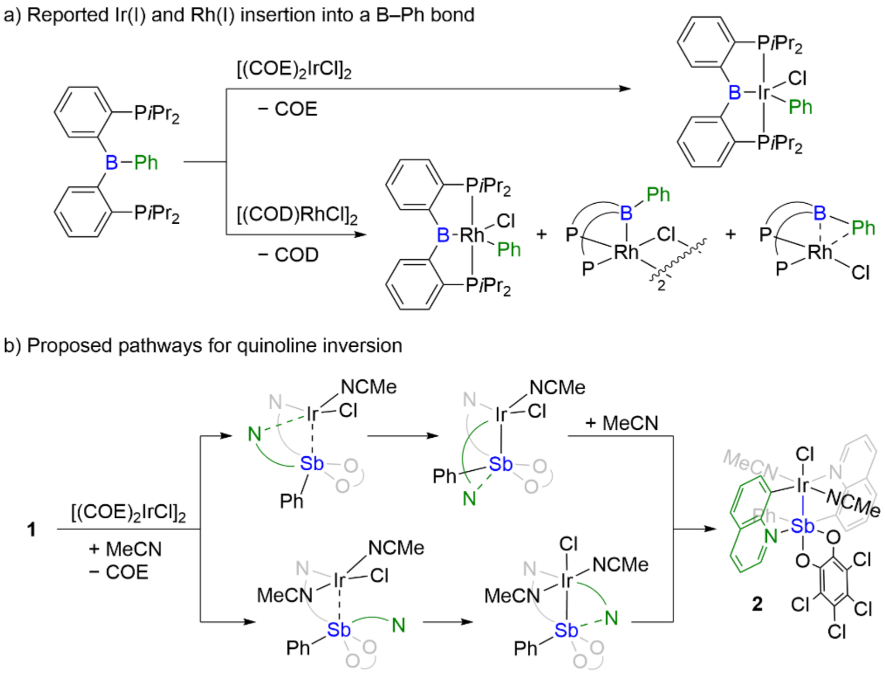

Scheme S1. a) Reported Ir(I) and Rh(I) insertion into a B–Ph bond. b) Proposed pathways for quinoline inversion in complex **2** via the insertion of Ir(I) into Sb–C bond.

7. Attempts for Reacting Sb(III) Proligand to Ir(I)

Initial attempts have been made to react Sb(III) proligand with Ir(I) precursor, however, no clean reaction or stable isolatable product was found.

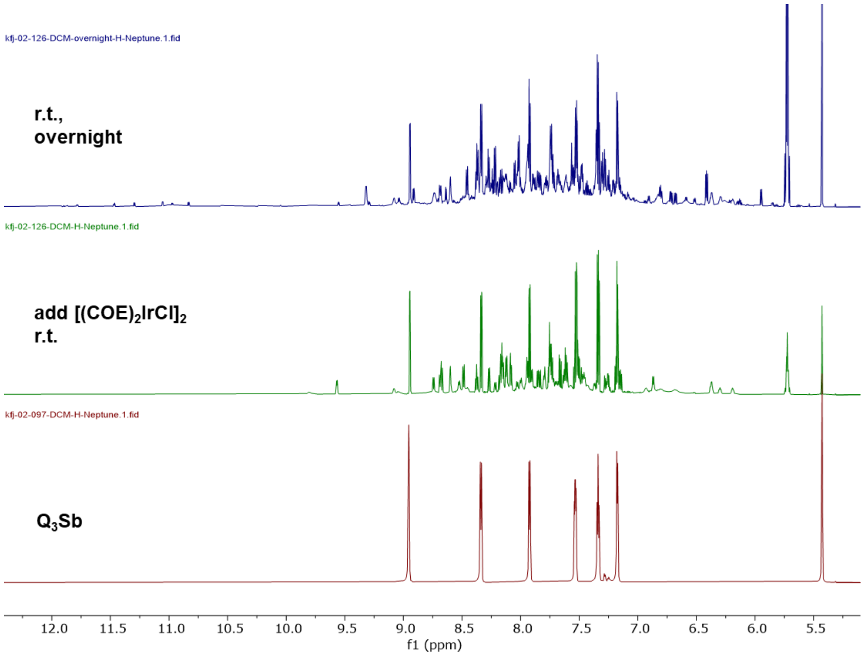

Figure S6. ^{1}H NMR spectra of reacting Q_3Sb with 0.5 eq. $[(COE)_2IrCl]_2$ (800 MHz, CD_2Cl_2).

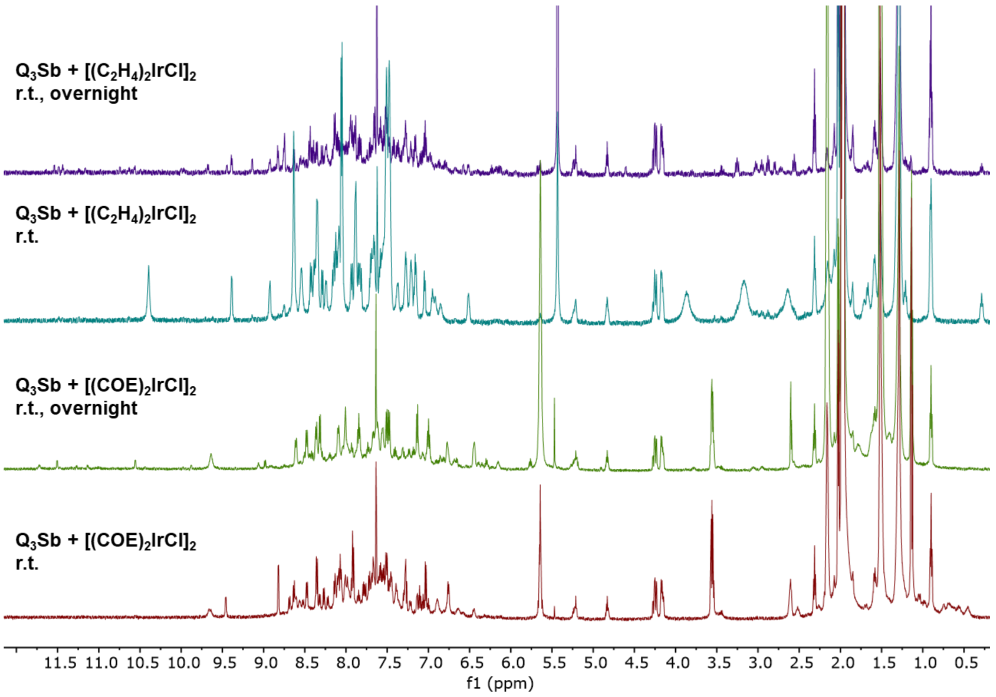

Figure S7. ^{1}H NMR spectra of reacting Q_3Sb with 0.5 eq. $[(COE)_2IrCl]_2$ or $[(C_2H_4)_2IrCl]_2$ (600 MHz, CD_3CN).

8. NMR Spectra

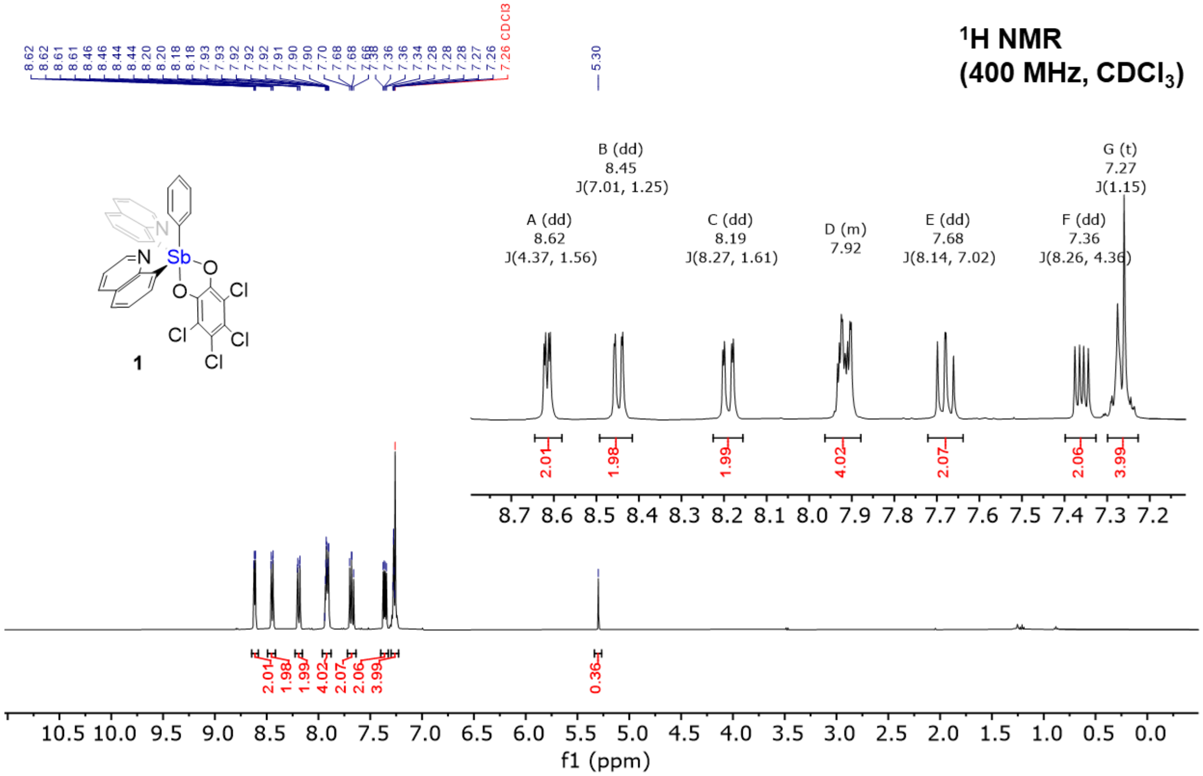

Figure S8. ^{1}H NMR spectrum of Q_2SbPh(*o*-chlor) (**1**) (400 MHz, $CDCl_3$).

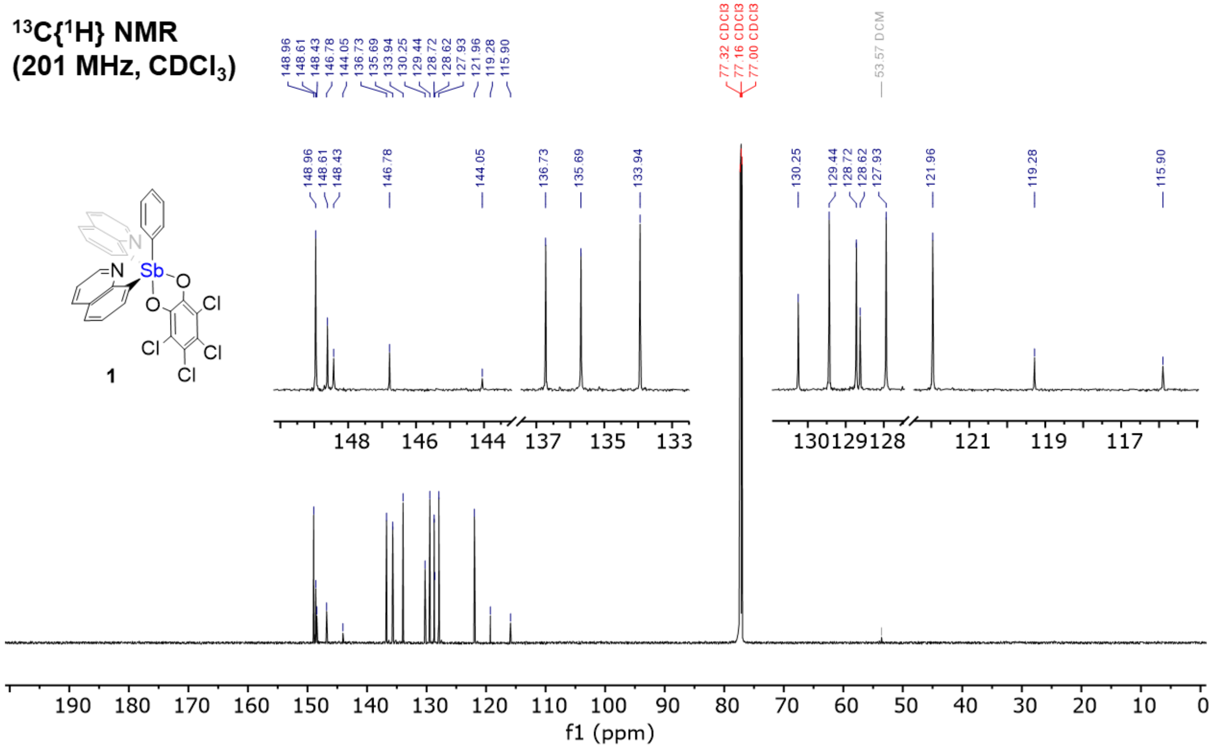

Figure S9. ^{13}C{^{1}H} NMR spectrum of Q_2SbPh(*o*-chlor) (**1**) (201 MHz, $CDCl_3$).

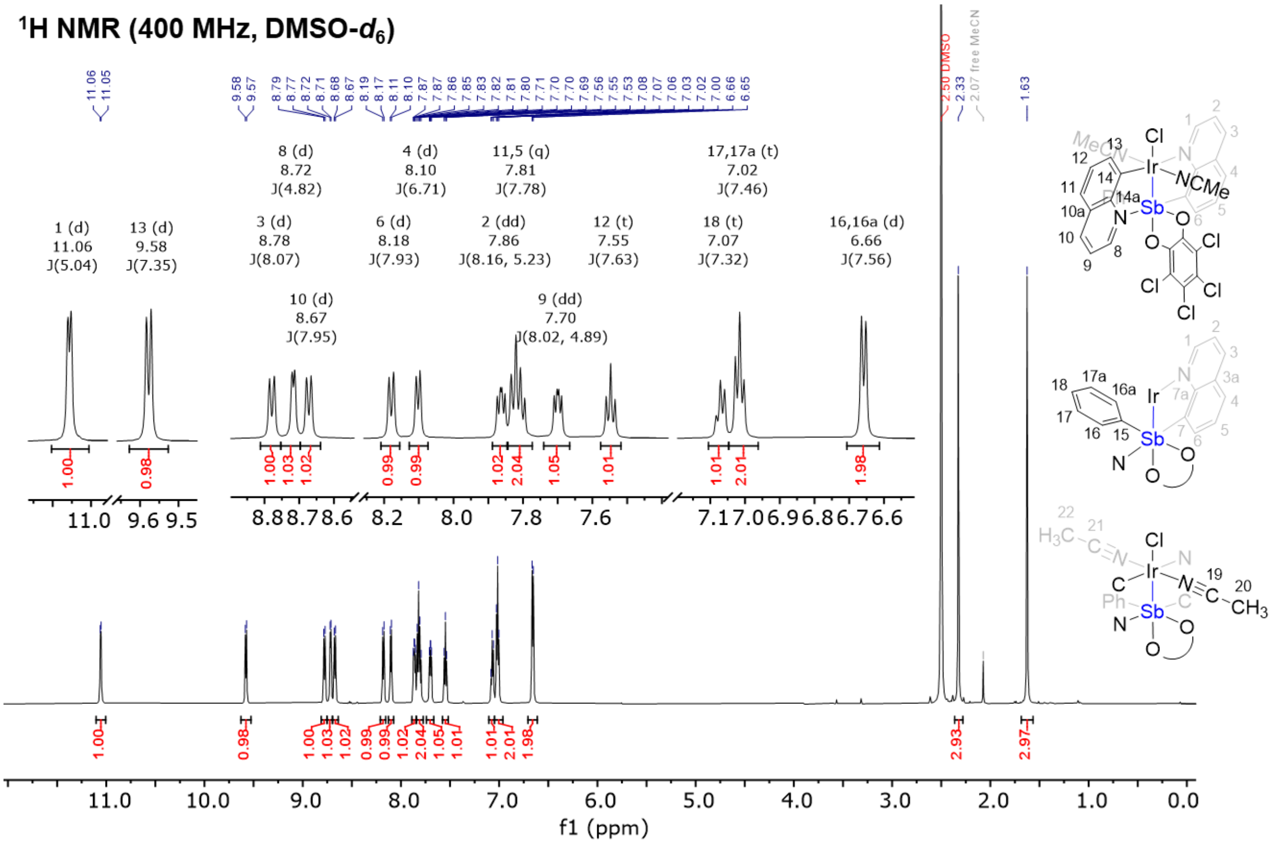

Figure S10. ^{1}H NMR spectrum of {Q_2SbPh(*o*-chlor)}Ir($NCMe)_2$(Cl) (**2**) (400 MHz, DMSO-d_6).

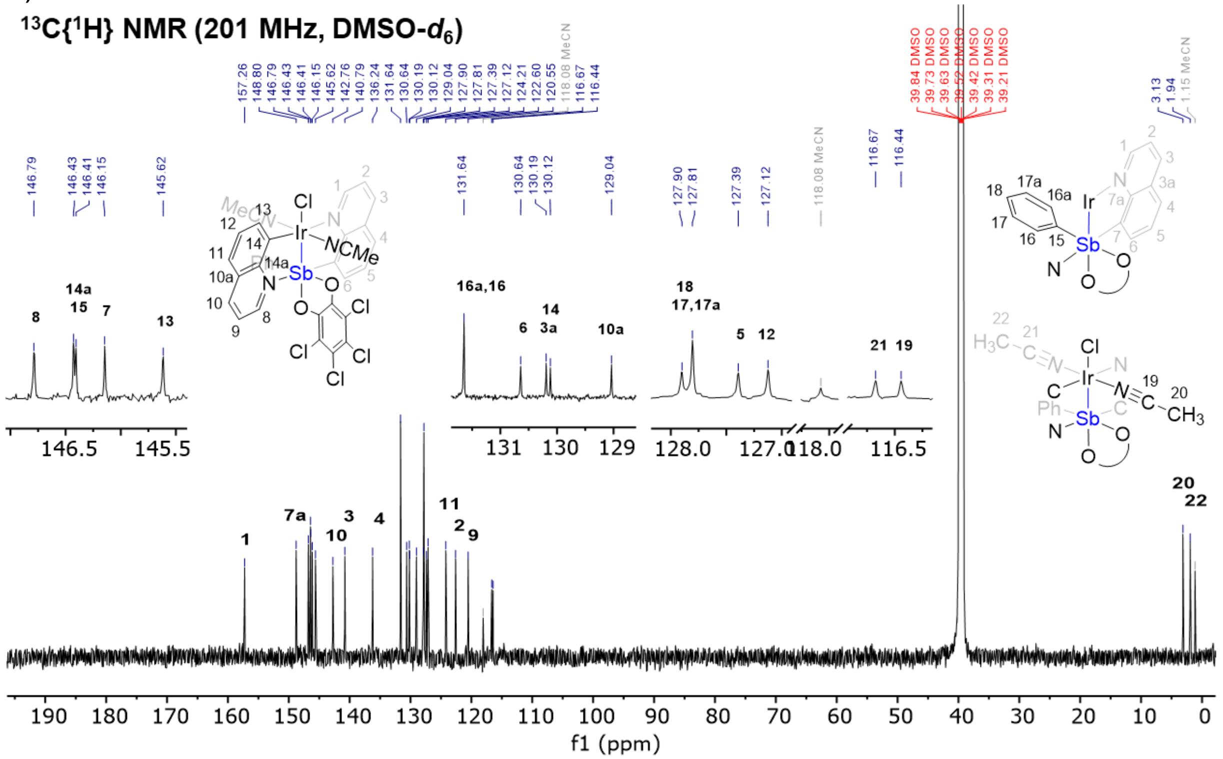

Figure S11. $^{13}C\{^1H\}$ NMR spectrum of complex **2** (201 MHz, DMSO-d_6).

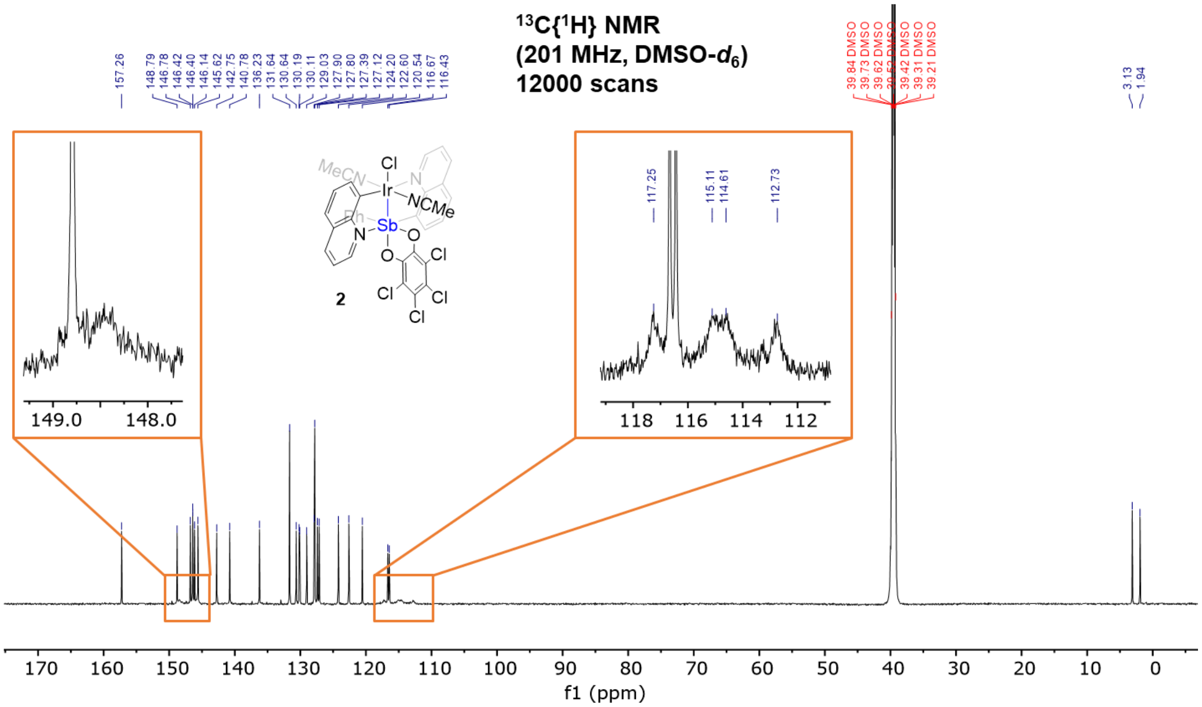

Figure S12. $^{13}C\{^1H\}$ NMR spectrum of complex **2** (201 MHz, DMSO-d_6). The zoomed in parts show the broad peaks belong to *o*-chloranil carbons.

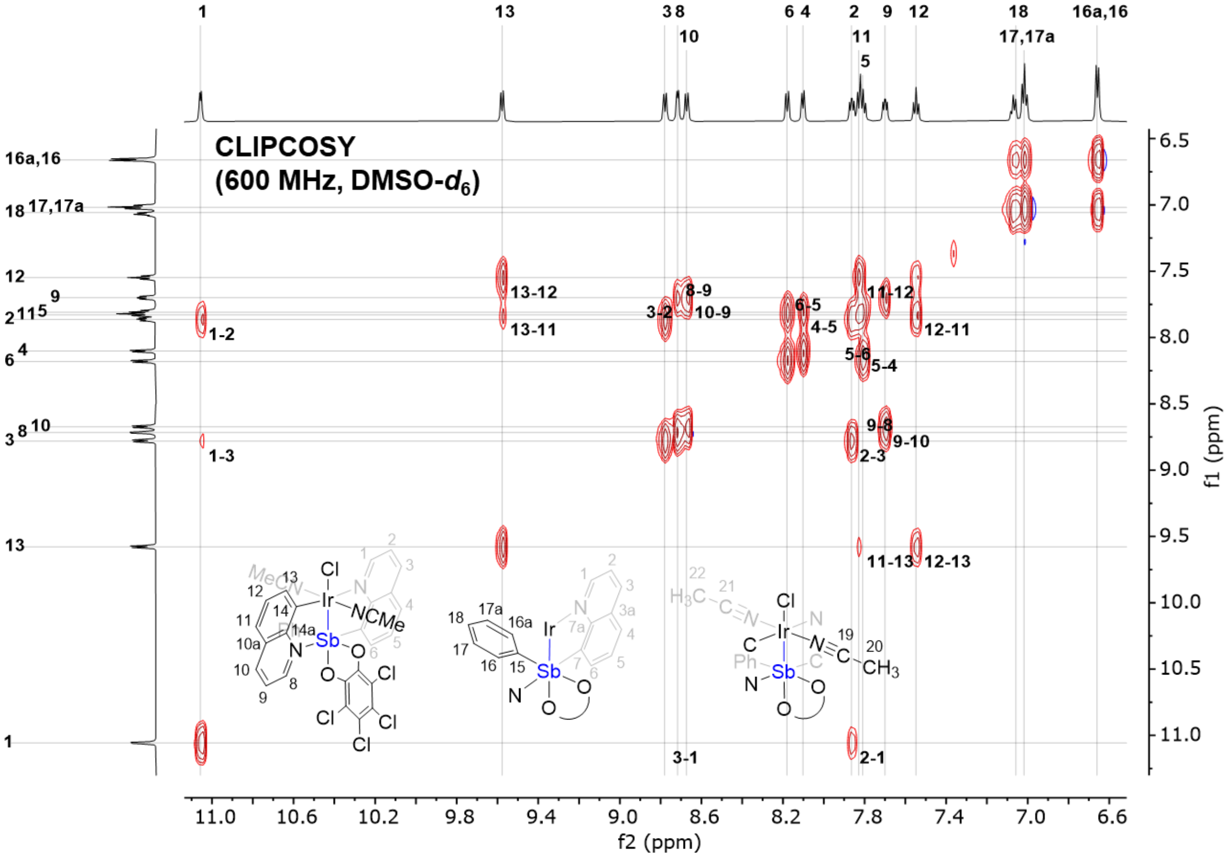

Figure S13. Clean in-phase COSY spectrum of complex **2** (600 MHz, DMSO-d_6).

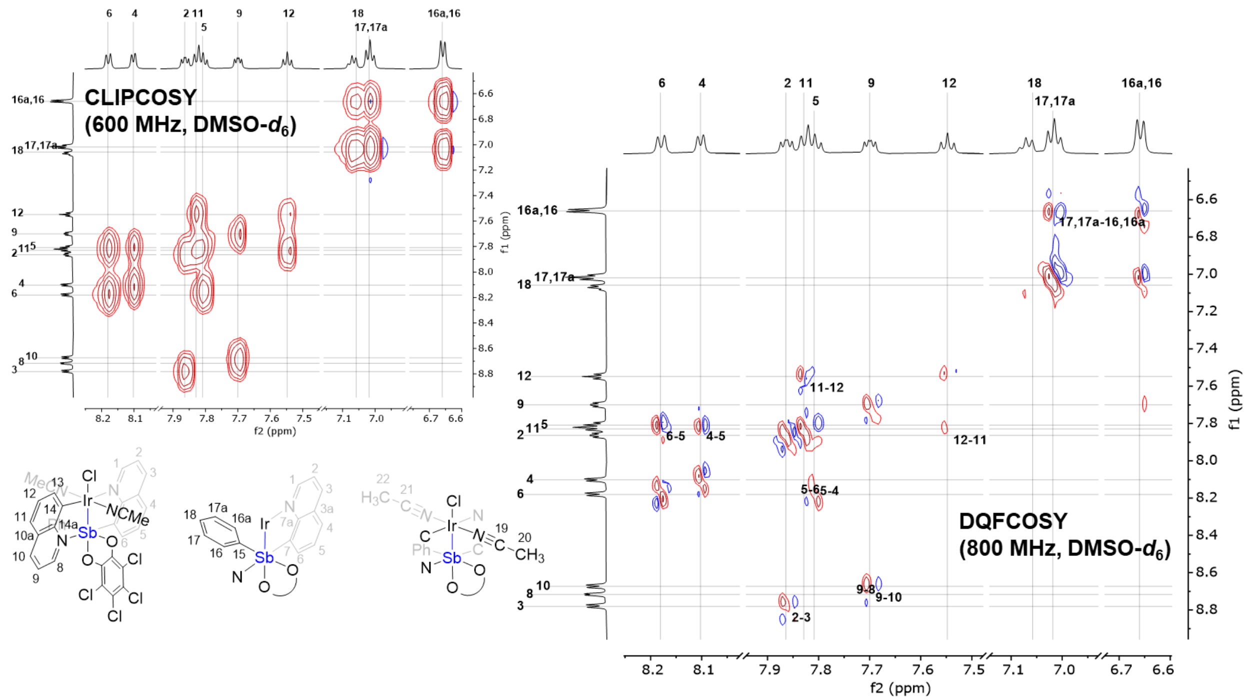

Figure S14. Zoomed in CLIPCOSY and double quantum filtered COSY (DQFCOSY) spectra of complex **2** (600 or 800 MHz, DMSO-d_6).

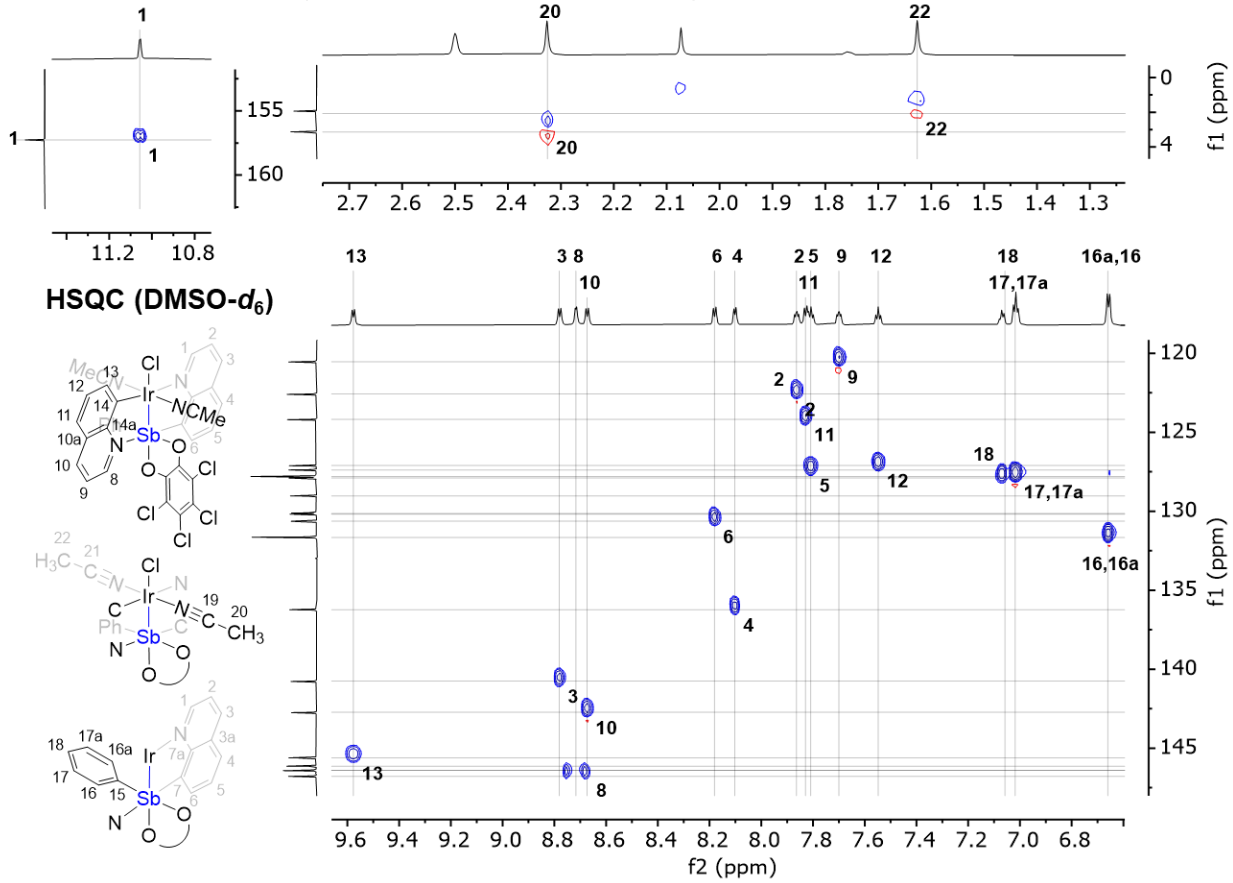

Figure S15. HSQC spectrum of complex **2** (800, 201 MHz, DMSO-d_6).

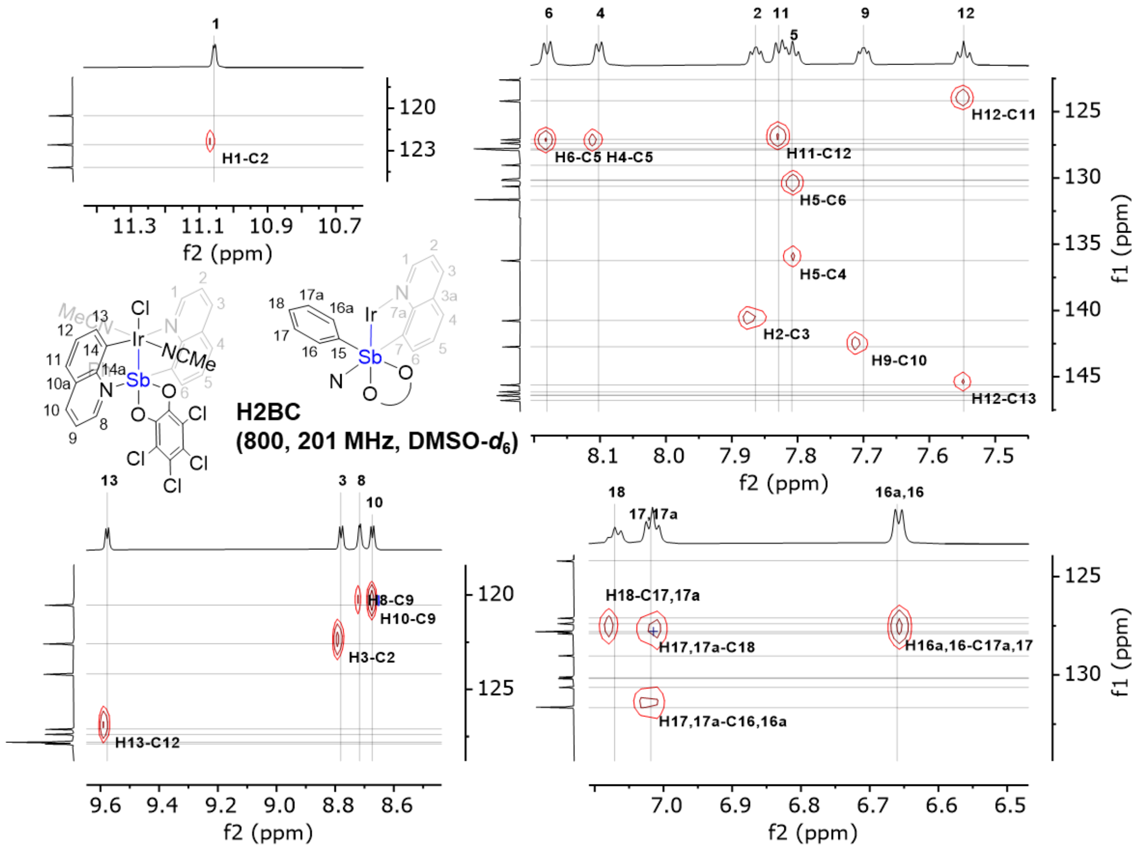

Figure S16. H2BC spectrum of complex **2** (800, 201 MHz, DMSO-d_6).

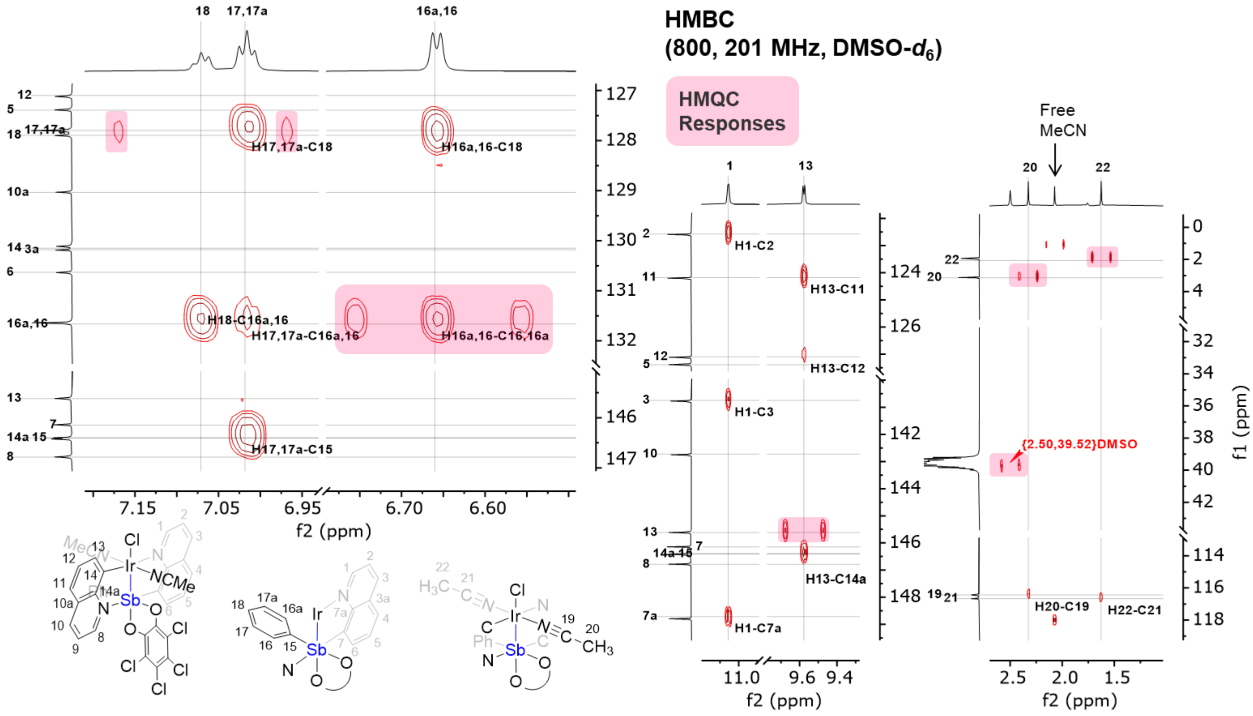

Figure S17. HMBC spectrum of complex **2** (800, 201 MHz, DMSO-d_6).

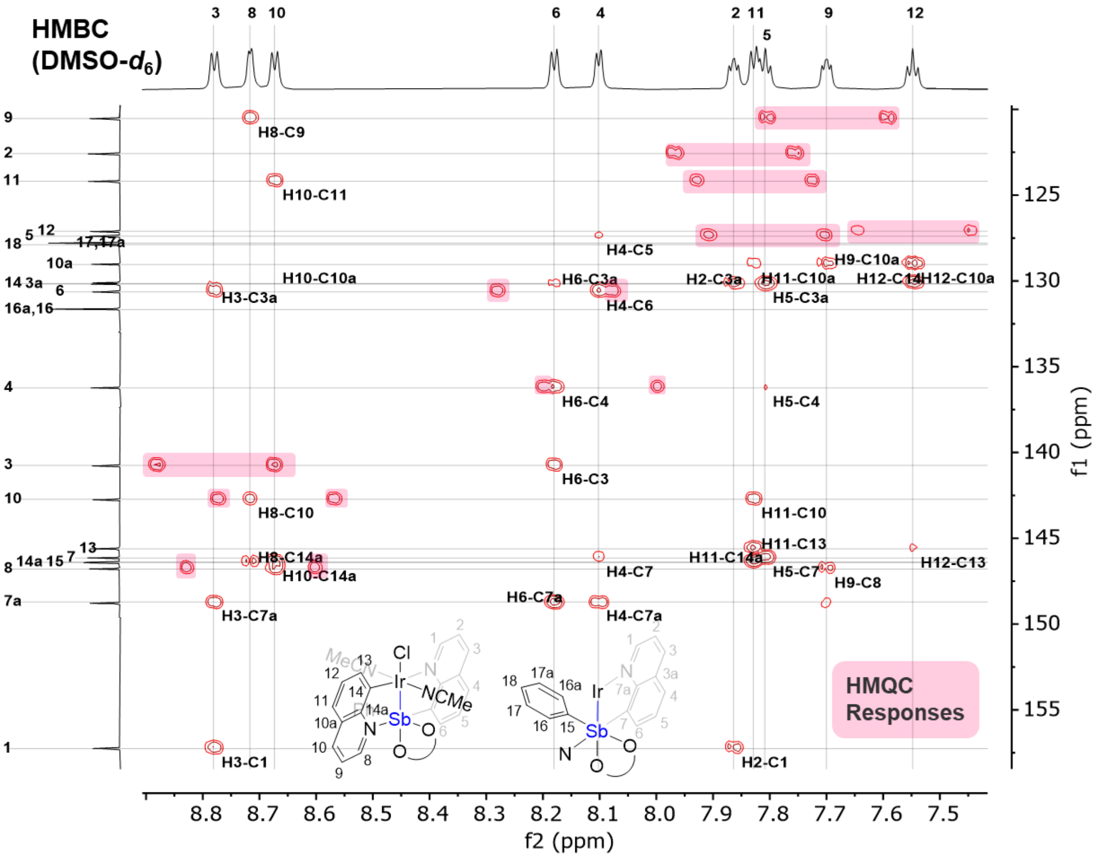

Figure S18. HMBC spectrum of complex **2** (800, 201 MHz, DMSO-d_6).

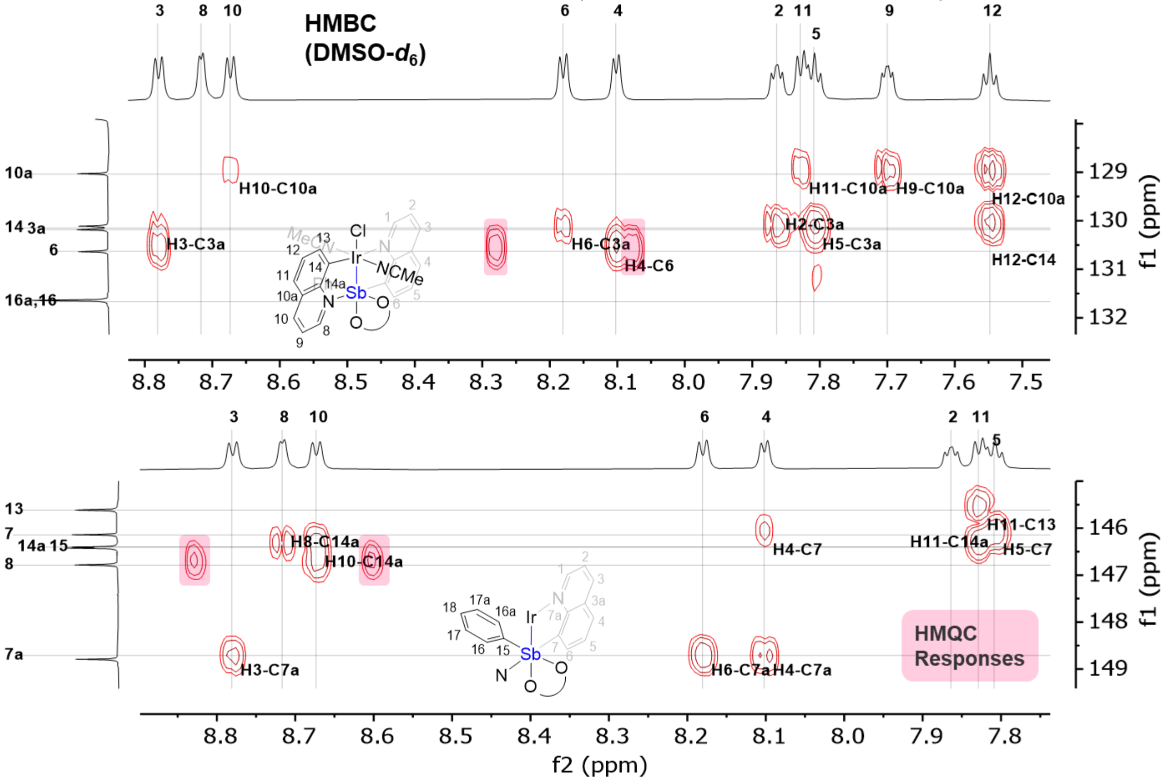

Figure S19. HMBC spectrum of complex **2** (800, 201 MHz, DMSO-d_6).

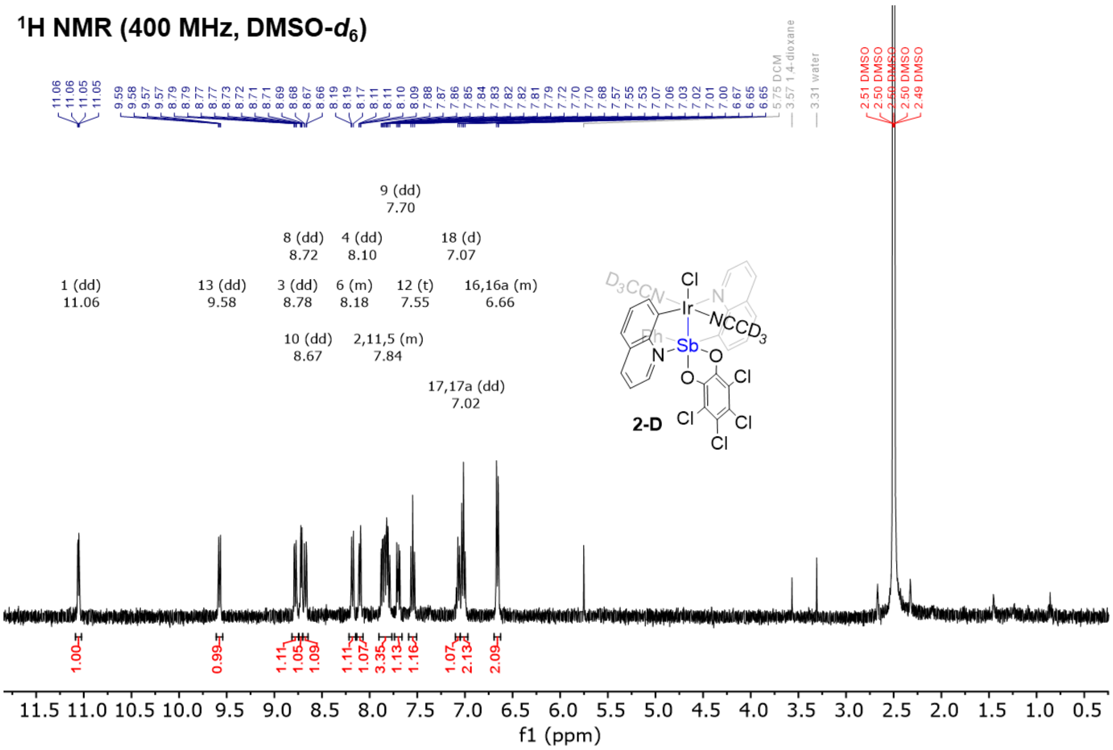

Figure S20. ^{1}H NMR spectra of {Q_2SbPh(*o*-chlor)}Ir($NCCD_3$)$_2$(Cl) (**2-D**) (400 MHz, DMSO-d_6).

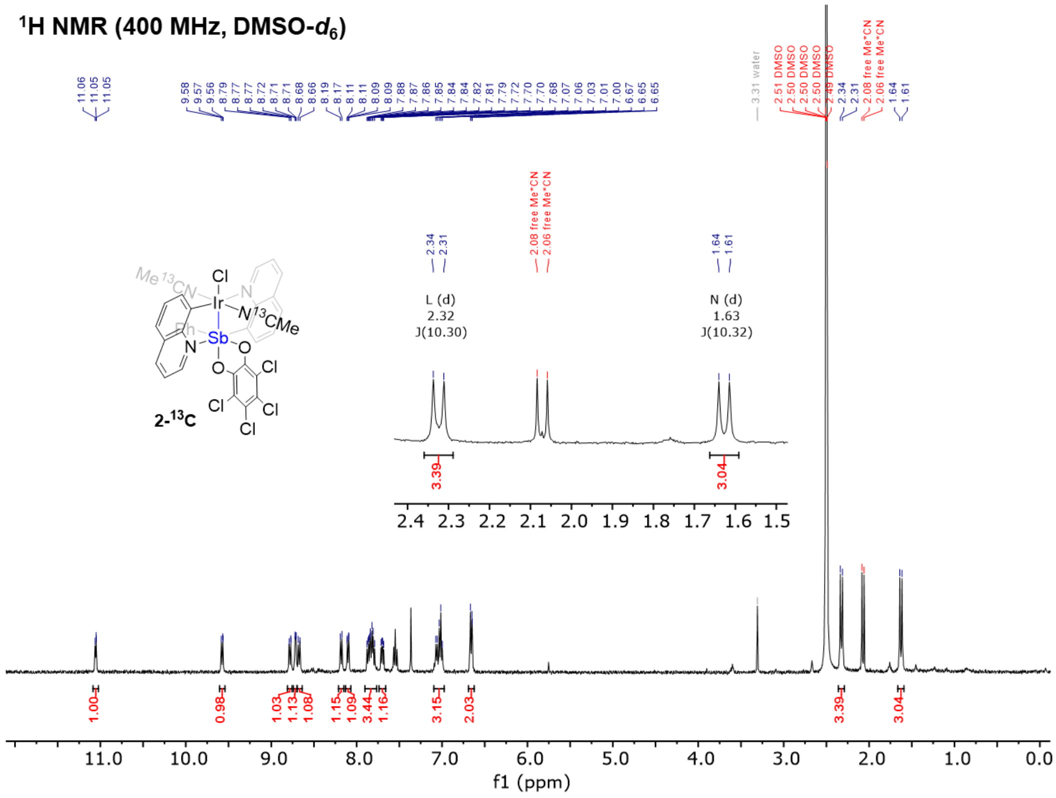

Figure S21. ^{1}H NMR spectrum of {Q_2SbPh(*o*-chlor)}Ir($N^{13}CMe$)$_2$(Cl) (**2-^{13}C**) (400 MHz, DMSO-d_6).

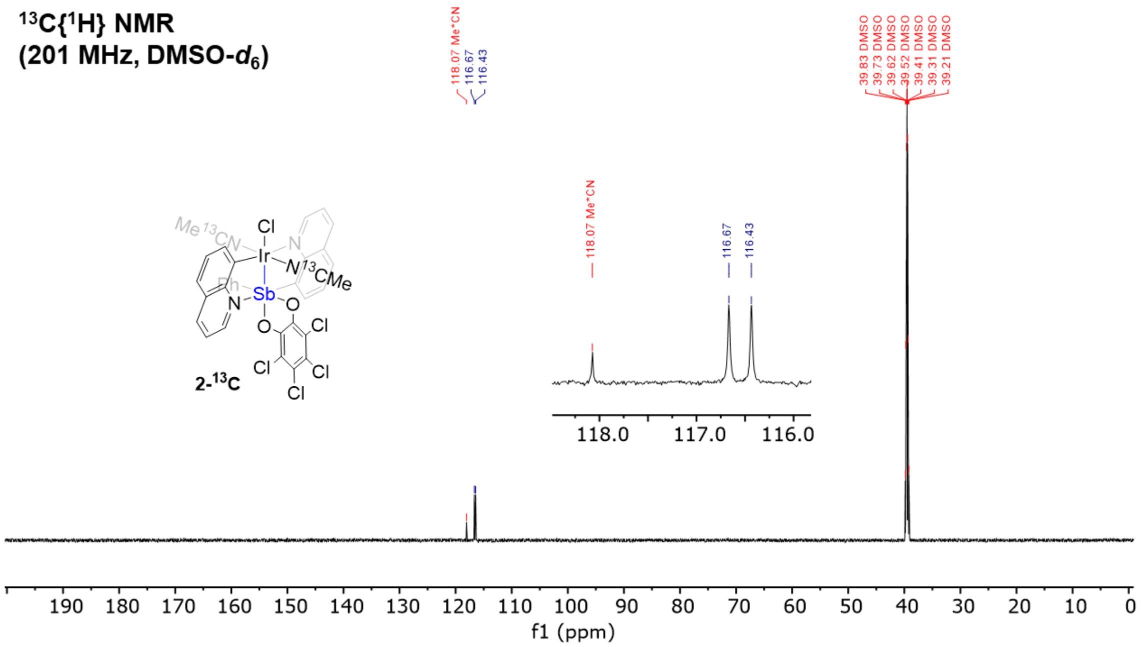

Figure S22. $^{13}C\{^{1}H\}$ NMR spectrum of complex **2-^{13}C** (400 MHz, DMSO-d_6).

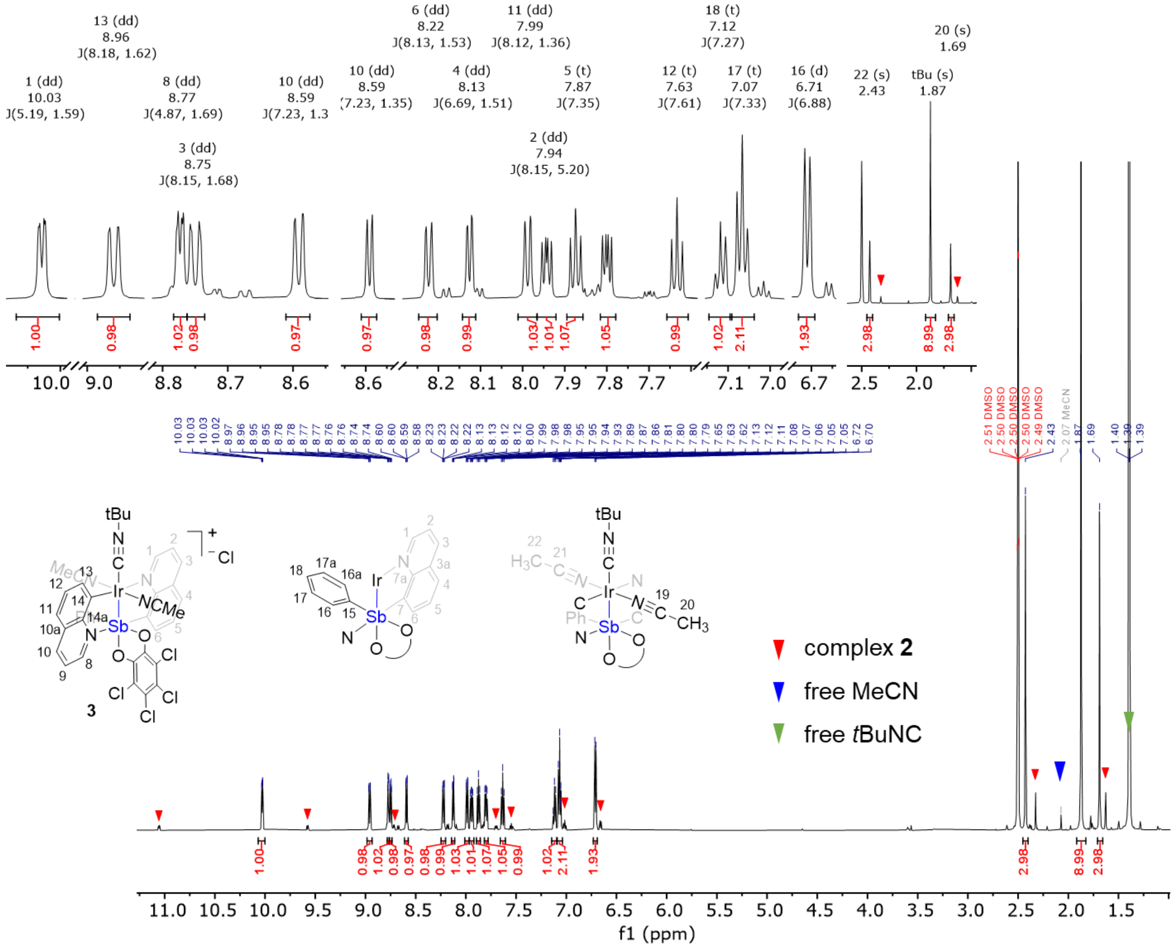

Figure S23. ^{1}H NMR spectrum of *in situ* formed [{Q_2SbPh(*o*-chlor)}Ir(NCMe)$_2$(*t*BuNC)]Cl (**3**) from the reaction of complex **2** with 2.0 eq. *t*BuNC (400 MHz, DMSO-d_6).

9. IR Spectra

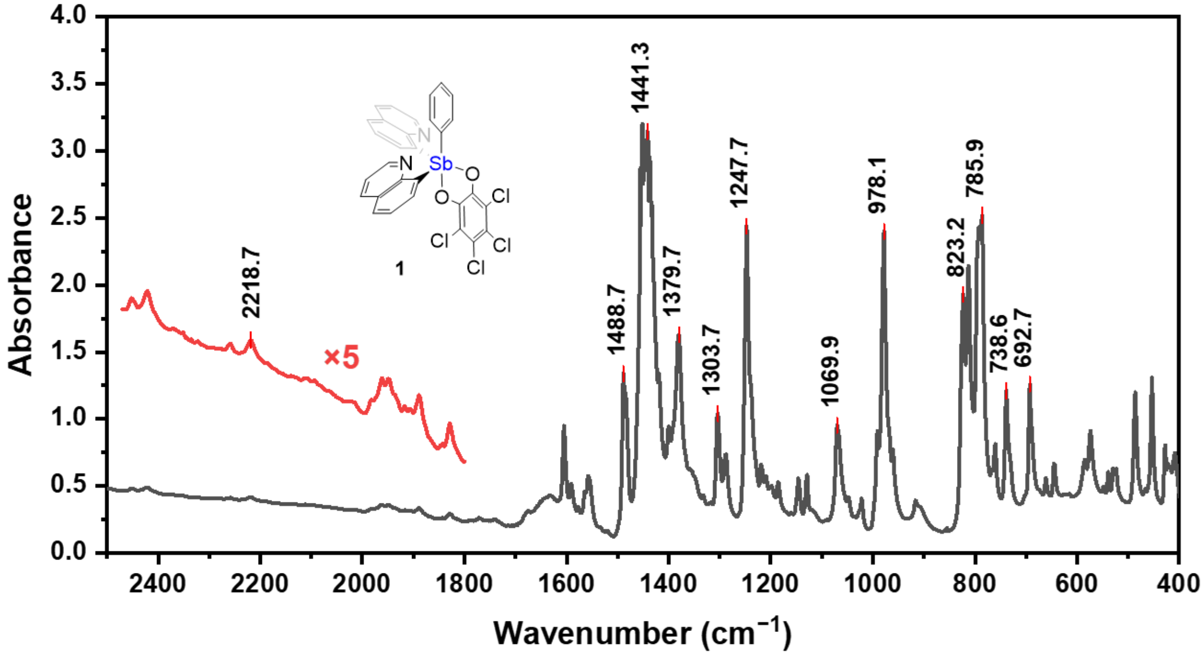

Figure S24. IR spectrum of Q_2SbPh(*o*-chlor) (**1**). The inset (in **red**) shows a 5× magnification of the low-intensity region of the spectrum.

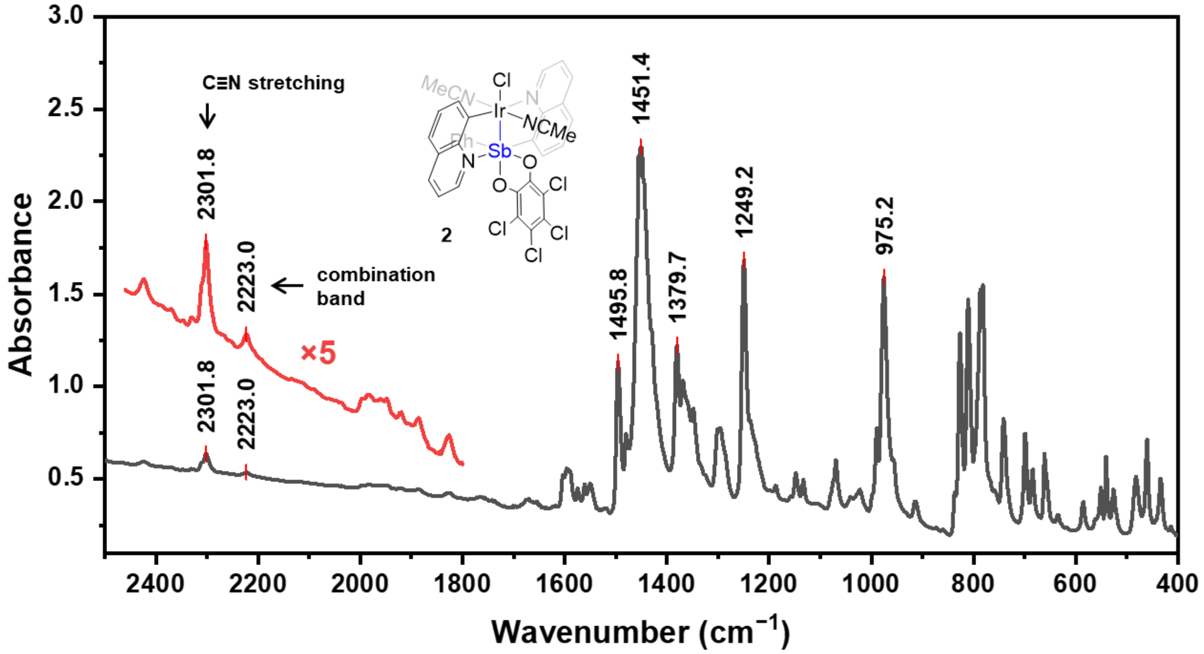

Figure S25. IR spectrum of {Q_2SbPh(*o*-chlor)}Ir(NCMe)$_2$(Cl) (**2**). The inset (in **red**) shows a 5× magnification of the low-intensity region of the spectrum.

The IR spectrum of complex **2** shows a weak band at 2302 cm^{-1} for the C≡N stretching (**Figure S26**, in **black**), which exhibits a significant blue-shift (49 cm^{-1}) compared to free MeCN as a neat liquid (ν_{CN} = 2253 cm^{-1}).[7] Sb–Ir chloride complexes with acetonitrile isotopologues, {Q_2SbPh(*o*-chlor)}Ir($NCCD_3$)$_2$(Cl) (**2-D**) and {Q_2SbPh(*o*-chlor)}Ir($N^{13}CMe$)$_2$(Cl) (**2-^{13}C**), were synthesized to further confirm the identity of the observed weak IR band. As presented in, the observed slightly shifted band for CD_3C≡N–Ir (2312 cm^{-1} in **red**), and the close match of experimentally observed ^{13}C≡N stretching (2255 cm^{-1}, in **blue**) with calculated frequency from ^{12}C≡N stretching in **2** based on the square root of reduced mass ratio (2254 cm^{-1}), further confirmed the IR band at 2302 cm^{-1} of **2** is designated as the C≡N stretching in complex **2**. The observed shifts for acetonitrile isotopologues are also consistent with previous IR studies.[8-9]

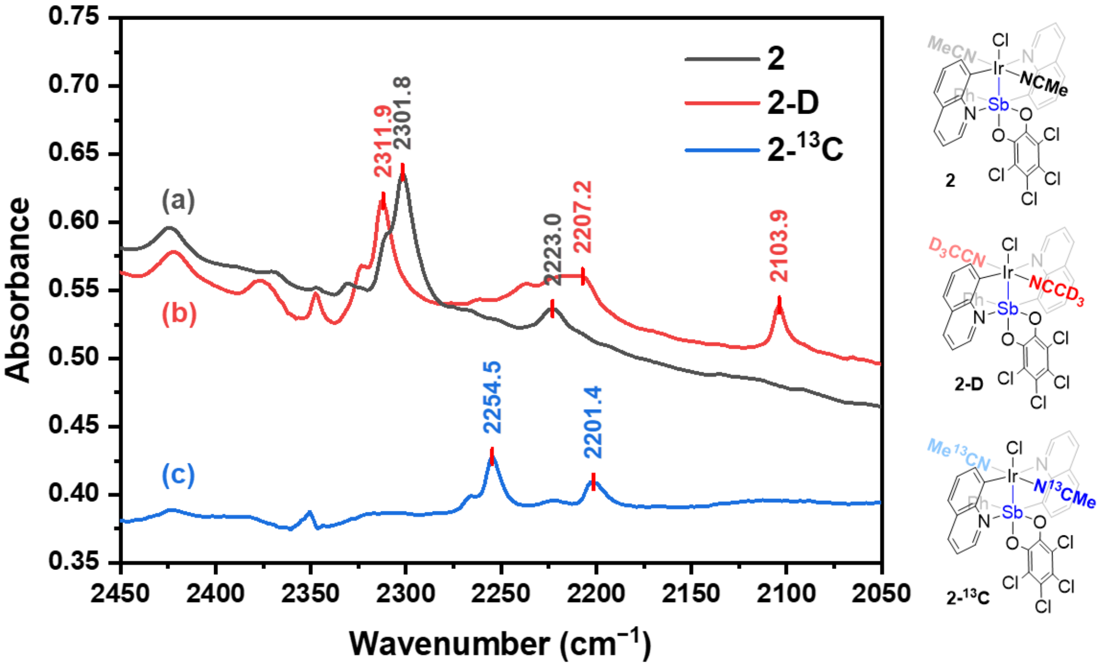

Figure S26. IR spectra of Sb–Ir chloride complexes with acetonitrile isotopologues: (a) {Q_2SbPh(*o*-chlor)}Ir(NCMe)$_2$(Cl) (**2**) in **black**. (b) {Q_2SbPh(*o*-chlor)}Ir($NCCD_3$)$_2$(Cl) (**2-D**) in **red**. (c) {Q_2SbPh(*o*-chlor)}Ir($N^{13}CMe$)$_2$(Cl) (**2-^{13}C**) in **blue**. *Note*: the band at 2223 cm^{-1} is the combination band of 1249 and 975 cm^{-1}; the bands at 2207 and 2104 cm^{-1} are from the C–D stretching; the band at 2201 cm^{-1} belongs to the residual free Me^{13}CN.

10. X-Ray Crystal Structure Data

Single crystals of all complexes were coated with Paratone oil and mounted on a MiTeGen MicroLoop. The X-ray intensity data for Q_2SbPh(*o*-chlor) (**1**) was measured on a Bruker Kappa APEXII Duo system equipped with an Incoatec Microfocus IμS (Cu K_α, λ = 1.54178 Å) and a multi-layer mirror monochromator. Data for {Q_2SbPh(*o*-chlor)}Ir$(NCMe)_2$(Cl) (**2**) and [{Q_2SbPh(*o*-chlor)}Ir$(NCMe)_2$(*t*BuNC)]Cl (**3**) were measured on a Bruker D8 VENTURE dual wavelength Mo/Cu Kappa four-circle diffractometer equipped with a PHOTON III detector. The Mo *Kα* radiation (λ = 0.71073 A) from an Incoatec IμS 3.0 microfocus sealed tube with a HELIOS double bounce multilayer mirror as monochromator was used.

Data collection and processing were done within the Bruker APEX5 (**1** and **2**) or APEX6 (**3**) software suite.[10] All data were integrated with the Bruker SAINT software using a narrow-frame algorithm. Data were corrected for absorption effects using a Multi-Scan method (SADABS).[10] Each structure was solved by dual methods with XT[11] and refined by full-matrix least-squares methods against F^2 using XL[12] within OLEX2.[13] All non-hydrogen atoms were refined anisotropically. Hydrogen atoms were placed in geometrically calculated positions with $U_{iso} = 1.2U_{equiv}$ of the parent atom ($1.5U_{equiv}$ for methyl). All the CIF files were generated using FinalCif.[14]

In complexes **1** and **3**, disordered solvent located in the crystal lattice could not be adequately modeled with or without restraints. Therefore, the solvent was accounted for using the Platon SQUEEZE method.[15] For **1**, a void space of 319 A^3 containing 80 electrons was found. This corresponds to 2 molecules of DCM in the ASU. For **3**, a void space of 462 A^3 containing 101 electrons was found. This corresponds to 2 molecules of

MeCN in the ASU. In complex **3**, disordered moieties were refined using bond lengths restraints and displacement parameter restraints.

Table S3. Crystal structure data table for Q_2SbPh(*o*-chlor) (**1**), {Q_2SbPh(*o*-chlor)}Ir(NCMe)$_2$(Cl) (**2**), and [{Q_2SbPh(*o*-chlor)}Ir(NCMe)$_2$(*t*BuNC)]Cl (**3**).

	**1**	**2**	**3**
CCDC number	2464018	2464019	2464020
Empirical formula	$C_{30}H_{17}Cl_4N_2O_2Sb$	$C_{38}H_{29}Cl_5IrN_6O_2Sb$	$C_{39}H_{32}Cl_5IrN_5O_2Sb$
Formula weight	701.01	1092.87	1093.89
Temperature [K]	100(2)	100	100.00
Wavelength [Å]	1.54178	0.71073	0.71073
Crystal size [mm^3]	0.087×0.138×0.197	0.106 × 0.183 × 0.269	0.02 × 0.155 × 0.235
Crystal habit	colorless plate	yellow block	yellow plate
Crystal system	orthorhombic	triclinic	triclinic
Space group	*P* na2$_1$	*P*-1	*P*-1
a [Å]	25.5309(7)	10.0729(3)	12.4096(8)
b [Å]	12.5434(4)	13.2216(4)	13.8101(10)
c [Å]	9.8005(3)	16.2501(6)	15.1083(9)
α [°]	90	110.8340(10)	70.705(2)
β [°]	90	98.5550(10)	68.693(2)
γ [°]	90	102.0980(10)	88.699(2)
Volume [Å^3]	3138.55(16)	1917.81(11)	2262.8(3)
Z	4	2	2
ρ_{calc} [g·cm^{-3}]	1.484	1.893	1.606
μ [mm^{-1}]	10.339	4.562	3.866
F(000)	1384	1056	1060
2θ range [°]	6.92 to 136.50	4.26 to 66.35	4.41 to 55.15
Index ranges	−30 ≤ h ≤ 30<br>−14 ≤ k ≤ 15<br>−11 ≤ l ≤ 11	−15 ≤ h ≤ 15<br>−20 ≤ k ≤ 19<br>−24 ≤ l ≤ 24	−16 ≤ h ≤ 16<br>−17 ≤ k ≤ 17<br>−18 ≤ l ≤ 19
Reflections collected	32582	77395	75644
Independent reflections	5413<br>[R_{int} = 0.0534]	14641<br>[R_{int} = 0.0481]	10427<br>[R_{int} = 0.0582]
Data / Restraints / Parameters	5413 / 1 / 352	14641 / 0 / 482	10427 / 75 / 515
Goodness-of-fit on F^2	1.025	1.037	1.014
Final *R* indexes [$I \geq 2\sigma(I)$]	R_1 = 0.0292<br>wR_2 = 0.0664	R_1 = 0.0283<br>wR_2 = 0.0518	R_1 = 0.0312<br>wR_2 = 0.0750
Final *R* indexes [all data]	R_1 = 0.0309<br>wR_2 = 0.0671	R_1 = 0.0391<br>wR_2 = 0.0548	R_1 = 0.0439<br>wR_2 = 0.0812
Largest peak/hole [eÅ^{-3}]	1.04/−0.69	1.26/−1.44	1.27/−1.52

11. Computational Details

For all geometry optimizations the M06/def2-SVP method and basis set was used employing Gaussian 16 with the default ultrafine integration. During geometry optimization solvent effects were incorporated using the conductor-like polarizable continuum model (CPCM) method for acetonitrile (MeCN). All of the stationary points were characterized as a minimum using vibrational frequency analysis. The calculated C≡N stretching frequencies in complex **2** and were found to be 2434 and 2418 cm^{-1} (not scaled for the specific functional or basis set), which is 46 and 30 cm^{-1} blue-shifted compared to the DFT calculated frequency of free MeCN (2388 cm^{-1}).

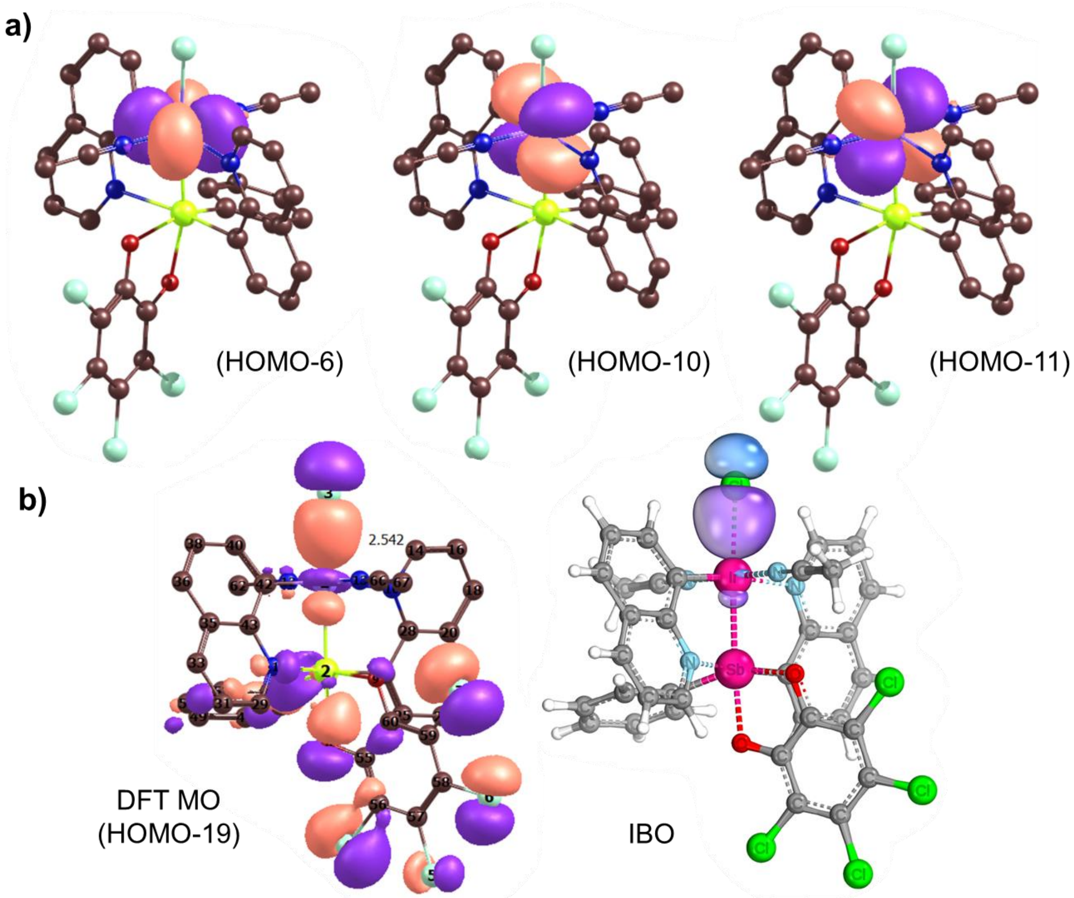

Figure S27.(a) Calculated Ir non-bonding orbitals. (b) Molecular orbital and IBO for the Ir–Cl interaction in complex **2**

The quantum theory of atoms in molecules (QTAIM) was used to analyze the bond critical point (BCP) between Ir and Sb in complex **2**, with the Multiwfn software package.[16-17] **Figure S28** shows a 2D contour map of the Laplacian of the electron density ($\nabla^2\rho$) with the Sb–Ir bond critical point (BCP) in blue, which indicates the presence of a bonding interaction between Sb and Ir. Delocalization index (δ), and H(r)/ρ point towards strong covalent/metallic bonding character. The Laplacian of the electron density at the BCP is indicative of metallic bonding. The singlet is the lowest in electronic energy (**Table S4**)

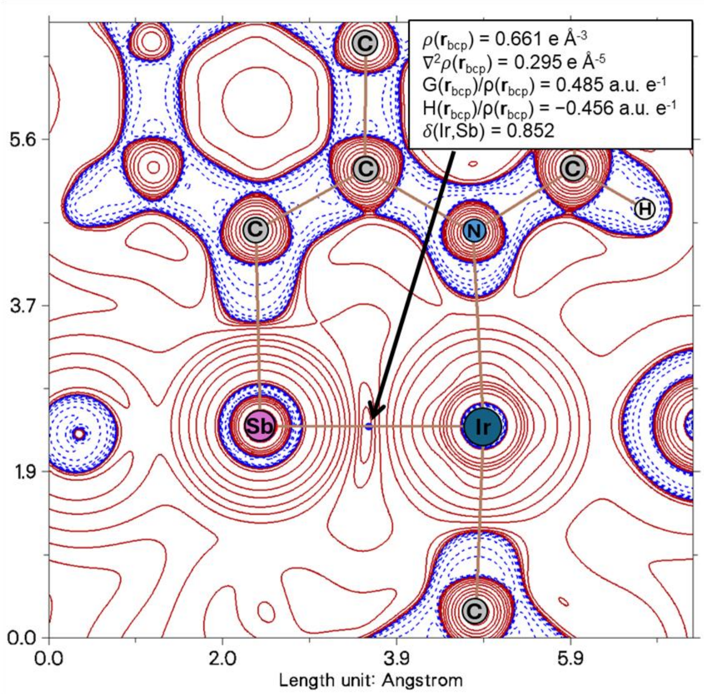

Figure S28. QTAIM analysis of the Sb–Ir bond in complex **2** including the BCP. For QTAIM, BCP is shown in blue, and in-plane bonds are shown in brown and plotted as $\nabla^2\rho$ with positive values in red (e^- depletion) and negative values in blue (e^- concentration).

Table S4. Energies of complex **2** at different spin states. All energies are in Hartree.

Complex	singlet	triplet	quintet
**2**	−4325.026986	−4324.943216	−4324.843709

12. References

1. Webber, C. K.; Kong, F.; Kumawat, J.; Joy, J.; Richardson, E. K.; Siano, P.; Dickie, D. A.; Ess, D. H.; Gunnoe, T. B., Synthesis of Quinoline-Based Pt–Sb Complexes with L- or Z-Type Interaction: Ligand-Controlled Redox via Anion Transfer. *Organometallics* **2024,** *43*, 1789-1802. doi: 10.1021/acs.organomet.4c00221

2. Onderdelinden, A. L.; van der Ent, A., Chloro- and bromo-(alkene)iridium(I) complexes. *Inorg. Chim. Acta* **1972,** *6*, 420-426. doi: 10.1016/S0020-1693(00)91830-9

3. Fulmer, G. R.; Miller, A. J. M.; Sherden, N. H.; Gottlieb, H. E.; Nudelman, A.; Stoltz, B. M.; Bercaw, J. E.; Goldberg, K. I., NMR Chemical Shifts of Trace Impurities: Common Laboratory Solvents, Organics, and Gases in Deuterated Solvents Relevant to the Organometallic Chemist. *Organometallics* **2010,** *29*, 2176-2179. doi: 10.1021/om100106e

4. Brown, S. N., Metrical Oxidation States of 2-Amidophenoxide and Catecholate Ligands: Structural Signatures of Metal–Ligand π Bonding in Potentially Noninnocent Ligands. *Inorg. Chem.* **2012,** *51*, 1251-1260. doi: 10.1021/ic202764j

5. Shih, W.-C.; Gu, W.; MacInnis, M. C.; Timpa, S. D.; Bhuvanesh, N.; Zhou, J.; Ozerov, O. V., Facile Insertion of Rh and Ir into a Boron–Phenyl Bond, Leading to Boryl/Bis(phosphine) PBP Pincer Complexes. *J. Am. Chem. Soc.* **2016,** *138*, 2086-2089. doi: 10.1021/jacs.5b11706

6. Jones, J. S.; Wade, C. R.; Gabbaï, F. P., Redox and Anion Exchange Chemistry of a Stibine–Nickel Complex: Writing the L, X, Z Ligand Alphabet with a Single Element. *Angew. Chem. Int. Ed.* **2014,** *53*, 8876-8879. doi: 10.1002/anie.201404156

7. Reimers, J. R.; Hall, L. E., The Solvation of Acetonitrile. *J. Am. Chem. Soc.* **1999,** *121*, 3730-3744. doi: 10.1021/ja983878n

8. Cho, H.-G.; Andrews, L., Infrared Spectra of CH3CN→M, M−η2-(NC)–CH3, CH3–MNC Prepared by Reactions of Laser-Ablated Fe, Ru, and Pt Atoms with Acetonitrile in Excess Argon. *Inorg. Chem.* **2019,** *58*, 16194-16204. doi: 10.1021/acs.inorgchem.9b02716

9. Cho, H.-G.; Andrews, L., Infrared Spectra of the Complexes Os←NCCH3, Re←NCCH3, CH3–ReNC, CH2═Re(H)NC, and CH≡Re(H)2NC and their Mn Counterparts Prepared by Reactions of Laser-Ablated Os, Re, and Mn Atoms with Acetonitrile in Excess Argon. *Organometallics* **2012,** *31*, 6095-6105. doi: 10.1021/om300456k

10. Bruker *Saint; SADABS; APEX.*, Bruker AXS Inc.: Madison, Wisconsin, USA., 2012.

11. Sheldrick, G. M., *SHELXT* – Integrated space-group and crystal-structure determination. *Acta Cryst. Sect. A Found. Adv.* **2015,** *71*, 3-8. doi: 10.1107/s2053273314026370

12. Sheldrick, G. M., Crystal structure refinement with *SHELXL*. *Acta Cryst. Sect. C Struct. Chem.* **2015,** *71*, 3-8. doi: 10.1107/s2053229614024218

13. Dolomanov, O. V.; Bourhis, L. J.; Gildea, R. J.; Howard, J. A. K.; Puschmann, H., OLEX2: a complete structure solution, refinement and analysis program. *J. Appl. Crystallogr.* **2009,** *42*, 339-341. doi: 10.1107/S0021889808042726

14. Kratzert, D. FinalCif. https://dkratzert.de/finalcif.html.

15. Spek, A. L., PLATON SQUEEZE: a tool for the calculation of the disordered solvent contribution to the calculated structure factors. *Acta Crystallogr. Sect. C: Struct. Chem.* **2015,** *71*, 9-18. doi: 10.1107/s2053229614024929

16. Lu, T., A comprehensive electron wavefunction analysis toolbox for chemists, Multiwfn. *J. Chem. Phys.* **2024,** *161*. doi: 10.1063/5.0216272

17. Lu, T.; Chen, F., Multiwfn: A multifunctional wavefunction analyzer. *J. Comput. Chem.* **2012,** *33*, 580-592. doi: 10.1002/jcc.22885
